# Antimalarials and Phytotoxins from *Botryosphaeria dothidea* Identified from a Seed of Diseased *Torreya taxifolia*

**DOI:** 10.3390/molecules26010059

**Published:** 2020-12-24

**Authors:** Mallika Kumarihamy, Luiz H. Rosa, Natascha Techen, Daneel Ferreira, Edward M. Croom, Stephen O. Duke, Babu L. Tekwani, Shabana Khan, N. P. Dhammika Nanayakkara

**Affiliations:** 1 National Center for Natural Products Research, Research Institute of Pharmaceutical Sciences, School of Pharmacy, The University of Mississippi, University, MS 38677, USA; ntechen@olemiss.edu (N.T.); btekwani@southernresearch.org (B.L.T.); skhan@olemiss.edu (S.K.); 2 Division of Pharmacognosy, Department of BioMolecular Sciences, School of Pharmacy, The University of Mississippi, University, MS 38677, USA; dferreir@olemiss.edu (D.F.); emcroom@olemiss.edu (E.M.C.J.); 3 Departamento de Microbiologia, Universidade Federal de Minas Gerais, Belo Horizonte, MG, Brazil; lhrosa@icb.ufmg.br; 4 Natural Products Utilization Research Unit, USDA-ARS, University, MS 38677, USA; sduke@olemiss.edu

**Keywords:** *Torreya taxifolia*, plant pathogenic fungi, *Botryosphaeria dothidea*, malaria, phytotoxin, *γ*-lactam alkaloids

## Abstract

The metabolic pathways in the apicoplast organelle of *Plasmodium* parasites are similar to those in plastids in plant cells and are suitable targets for malaria drug discovery. Some phytotoxins released by plant pathogenic fungi have been known to target metabolic pathways of the plastid; thus, they may also serve as potential antimalarial drug leads. An EtOAc extract of the broth of the endophyte *Botryosphaeria dothidea* isolated from a seed collected from a *Torreya taxifolia* plant with disease symptoms, showed in vitro antimalarial and phytotoxic activities. Bioactivity-guided fractionation of the extract afforded a mixture of two known isomeric phytotoxins, FRT-A and flavipucine (or their enantiomers, sapinopyridione and (-)-flavipucine), and two new unstable *γ*-lactam alkaloids dothilactaenes A and B. The isomeric mixture of phytotoxins displayed strong phytotoxicity against both a dicot and a monocot and moderate cytotoxicity against a panel of cell lines. Dothilactaene A showed no activity. Dothilactaene B was isolated from the active fraction, which showed moderate in vitro antiplasmodial activity with high selectivity index. In spite of this activity, its instability and various other biological activities shown by related compounds would preclude it from being a viable antimalarial lead.

## 1. Introduction

Parasites of the genus *Plasmodium*, which cause malaria, contain an organelle called the apicoplast, and its functioning is essential for the survival in both the erythrocytic and the hepatic phases of development in mammalian hosts [1]. The apicoplast is similar to plastids of plants, as it is thought to be a vestigial plastid derived from endosymbiosis of a red alga by a heterotropic, unicellular eukaryote [2]. It retains plant-like metabolic pathways, which are absent in vertebrate hosts, making the enzymes of these pathways suitable targets for malaria drug discovery [3,4,5,6]. Some phytotoxins released by plant pathogenic fungi inhibit metabolic pathways of the plastid [7]. If the compounds produced by plant pathogenic fungi show phytotoxicity and malarial parasite death without causing cytotoxicity towards mammalian cells, it indicates that the mechanism of parasitic death may be due to the ability of the compounds to inhibit the plant-like metabolic pathways in the apicoplast. As part of our program to search for new antimalarials from plant pathogenic fungi [8,9,10,11,12], we investigated fungi from seeds of a diseased *Torreya taxifolia* Arnott. (Taxaceae). *T. taxifolia*, also known as Florida nutmeg, Florida torreya, stinking-cedar, or gopherwood, is a rare, critically endangered evergreen conifer endemic to three counties in Northern Florida [13,14,15]. The decline of the native population during the recent past has been attributed to both abiotic and biotic causes, including fungal diseases. Several fungi have been isolated from diseased *T. taxifolia* and some of them have been shown to cause leaf spots and canker disease in healthy plants [15,16,17,18]. For this study, seeds of *T. taxifolia* were collected from a tree with disease symptoms cultivated on the Biltmore Estate in Asheville, North Carolina.

From fragments of a surface-sterilized seed, several endophytic fungi were isolated. An EtOAc extract of the broth of one of these fungi grown in potato-dextrose liquid medium showed phytotoxic and antiplasmodial activities. This fungus (UM124) was identified as *Botryosphaeria dothidea* (*Botryosphaeriaceae*) by DNA analysis. Members of the family *Botryosphaeriaceae* (*Botryosphaeriales*, *Ascomycota*) cause leaf spots, fruit and root rots, and cankers in a variety of hosts [19], and *B. dothidea* has specifically been isolated from a large number of diseased and healthy woody plants, including many economically important crops [20]. A *Botryosphaeria* sp. strain has previously been isolated from *T. taxifolia* leaves infected with needle-spot disease [14]. A chemical investigation of endophytic *B. dothidea* has previously been carried out, and a variety of compounds, including a simple α-pyridone, were reported from a solid culture of this fungus [21].

**Figure 1:**
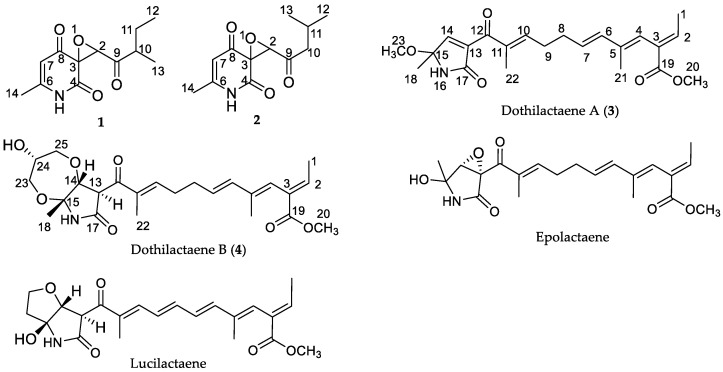
Structures of compounds.

Bioassay-guided fractionation of the active EtOAc extract resulted in the isolation and identification of a mixture of known, isomeric phytotoxins, FRT-A (**1**) [22] and flavipucine (**2**) [23] (or their enantiomers, sapinopyridione [24] and (-)-flavipucine [22]), as well as two new unstable *α*-alkyl-*γ*-lactam alkaloids, dothilactaenes A (**3**) and B (**4**), closely related to epolactaene [25] (Figure 1). While dothilactaene A showed no activity, dothilactaene B was isolated from the active fraction, which showed moderate, selective antiplasmodial activity. Two other components isolated from this fraction had the same molecular formula and similar NMR data indicating that they were diastereomers but due to the lack of sufficient materials their structural investigation was not completed. This is the first report of the isolation of a fungus producing phytotoxins from the seeds of diseased *T. taxifolia. B. dothidea* might play a significant role in decreasing the population of the endangered *T. taxifolia*.

## 2. Results and Discussion

The EtOAc extract of the fermentation broth of a fungus isolated from surface sterilized seed fragments of diseased *T. taxifolia* showed good phytotoxic activity against model plants, a dicot (lettuce, *Lactuca sativa* L.) and a monocot (bentgrass, *Agrostis stolonifera* L.). Furthermore, it showed good antiplasmodial activity against chloroquine-sensitive (D6) and -resistant (W2) strains of *Plasmodium falciparum* (IC_50_ = 0.86 and 1.3 µg/mL, respectively), with low cytotoxicity (32 µg/mL) to mammalian kidney fibroblasts (Vero cells).

The analysis of the ITS genomic region of UM124 for a closest neighbor with published sequences showed that the highest identity was with various strains of the species *Botryosphaeria dothidea*. Analysis of 18S rDNA of the fungus gave 100% sequence identity to *B. dothidea.* The construction of the phylogenetic tree with different strains of *B. dothidea* involved 29 nucleotide sequences with a total of 488 positions in the final dataset. In addition, for the construction of the phylogenetic tree of UM124 with various taxa of the family *Botryosphaeriaceae*, 92 nucleotide sequences were analyzed with a total of 382 positions in the final dataset (Supplementary Materials: Tables S1 and S2 and Figures S1 and S2).

The EtOAc extract of the culture broth was fractionated by silica gel column chromatography and the active fractions were combined and separated by Sephadex LH-20 gel filtration using MeOH as the mobile phase. A fraction with high phytotoxic activity and no antiplasmodial activity afforded a white precipitate, and its NMR data (Table 1) indicated that it was a 3:1 mixture of two isomeric known 2,4-pyridione epoxides, fruit rot toxin A (FRT-A) (**1**) [22,23] and (+)-flavipucine [24] (or their enantiomers, sapinopyridione [25] and (-)-flavipucine [24,26,27]). FRT-A was previously reported from *B. berengeriana* [23].

The subfraction with antiplasmodial activity (IC_50_ = 0.68 and 0.78 µg/mL) was further separated by reverse-phase semi-preparative HPLC to afford an inactive and an active fraction (IC_50_ < 0.523 µg/mL), with no cytotoxicity to Vero cells. The NMR data of the inactive fraction showed that it was more than 85% of a single compound with lipid impurities. Due to instability and limited material, further purification of this compound was not possible and was identified as dothilactaene A (**3**). The active fraction was separated by analytical HPLC to three peaks. The NMR data of the first peak showed it was a single compound with 90% purity with lipid contaminants and was identified as dothilactaene B (**4**).

The molecular weight of dothilactaene A (**3**) was determined as C_22_H_29_NO_5_ by HRESIMS. The ^1^H- and ^13^C-NMR data of **3** (Table 2) indicated the presence of 22 carbon resonances that consisted of five quaternary, three carbonyl, six methine, two methylene, and six methyl carbons. Analysis of the ^1^H- and ^13^C-NMR data indicated that it was an *α*-alkyl-*γ*-lactam derivative, related to epolactaene [28] (Figure 1). Comparison of the ^1^H and ^13^C-NMR data of these compounds (Table 2) showed that they had the same side chain but differed in the 2-pyrrolidone ring. These differences were attributable to the replacement of the epoxide and the hydroxy groups in the 2-pyrrolidone ring of epolactaene by a double bond and methoxy group, respectively, in **3**. In the HMBC spectrum of **3**, cross-peaks of H_3_-1 (*δ*_H_ 1.72) with C-2 (*δ*_C_ 139.9) and C-3 (*δ*_C_ 130.3); H-2 (*δ*_H_ 6.96) with C-3 (*δ*_C_ 130.3) and C-19 (*δ*_C_ 167.8); H-4 (*δ*_H_ 5.97) with C-21 (*δ*_C_ 14.3), C-19 (*δ*_C_ 167.8), and C-2 (*δ*_C_ 139.9); H_3_-21 (*δ*_H_ 1.62) with C-6 (*δ*_C_ 135.6) and C-4 (*δ*_C_ 123.1); H_2_-8 (*δ*_H_ 2.32) with C-6 (*δ*_C_ 135.6), C-7 (*δ*_C_ 128.2), C-9 (*δ*_C_ 28.4), and C-10 (*δ*_C_ 148.6); and H_3_-22 (*δ*_H_ 1.89) with C-10 (*δ*_C_ 148.6) and C-12 (*δ*_C_ 191.4) confirmed the structure of the side chain. Further, HMBC correlations of the -OCH_3_ singlet (*δ*_H_ 3.18) with C-15 (*δ*_C_ 89.2); the 18-CH_3_ singlet (*δ*_H_ 1.61) with C-15 (*δ*_C_ 89.2) and C-14 (*δ*_C_ 149.5); and the H-14 olefinic proton singlet (*δ*_H_ 6.83) with C-12 (*δ*_C_ 191.4), C-13 (*δ*_C_ 139.1), and C-17 (*δ*_C_ 168.3) confirmed the structure of the 2-pyrrolidone moiety and its link to the side chain. Other COSY and HMBC correlations supported this structure. The (*E*)-configuration of the Δ^6(7)^ double bond was determined by the coupling constant (*J* = 15.2 Hz). ROESY data indicated that the Δ^2(3)^, Δ^4(5)^ and Δ^10(11)^ olefinic bonds were all (*E*) configured.

**Figure 2:**
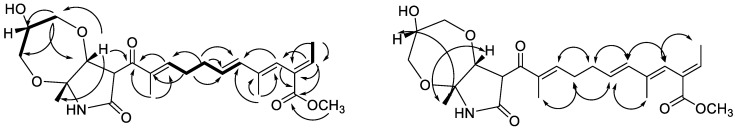
HMBC (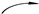) and COSY (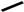) correlations of **4** ROESY (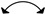) correlations of **4**.

Dothilactaene B (**4**) had the molecular formula, C_24_H_33_NO_7_, by HRESIMS data. Its NMR spectra showed resonances due to the same side chain as **3,** and COSY and HMBC data provided confirmatory evidence (Figure 2). Comparison of the remaining resonances with those of **3** indicated the absence of the olefinic bond and the methoxy group in the 2-pyrrolidone ring and the presence of three oxygenated carbons due to a glycerol moiety. The COSY and HMBC correlations (Figure 2) of **4** showed, respectively, an oxygenated methine (H-24; *δ*_H_ 3.65) coupled with two oxygenated methylenes (H_2_-23; *δ*_H_ 3.45, 3.49, and H_2_-25; *δ*_H_ 3.39, 3.58), and the methylene resonance at *δ*_H_ 3.39 (H-25) correlating with C-23 (*δ*_C_ 60.6) and C-24 (*δ*_C_ 70.4), confirming the presence of a disubstituted glycerol moiety. Even though it is not common, secondary metabolites with glycerol moieties have previously been isolated from endophytic fungi [29,30,31,32]. In the HMBC spectrum, H-14 (*δ*_H_ 4.29) had a cross peak with the oxygenated methylene at *δ*_C_ 60.7 (C-23) of glycerol, indicating one of its linkages. To satisfy the molecular formula C_24_H_33_NO_7_ and the index of hydrogen deficiency, there should be another ether linkage to the 2-pyrrolidone moiety.

Even though no other HMBC cross peaks were visible between the glycerol moiety and the 2-pyrrolidone ring, this link should most probably be between the remaining primary hydroxy group of the former and OH-15 of the latter. An HMBC cross peak between H-14 (*δ*_H_ 4.29) and the C-12 carbonyl (*δ*_C_ 196.2) confirmed the link between the 2-pyrrolidone ring and the side chain. Large coupling (*J* = 15.6 Hz) between H-6 and H-7 showed that the Δ^6(7)^ olefinic bond was in an *E* (*trans*) configuration. ROESY correlations (Figure 2) indicated the (*E*) configuration for the other double bonds in the side chain and the cofacial orientation of CH_3_-18, H-14, and H-24. Comparison of ^3^*J*_13,14_ value of **4** with those reported for fusarin A [33] and lucilactanen [34,35] indicates an *anti* configuration of H-13/H-14. These data permitted assignment of the (13S*, 14R*, 15R*, 24R*) relative configuration. However, the limited sample quantity and the instability precluded electronic circular dichroism (ECD) studies to assign its absolute configuration.

The active fraction afforded two additional components, which had the same molecular formula, C_24_H_33_NO_7_, as that of **4**. NMR data analysis (Supplementary Materials) showed that they have the same gross structure but differ from **4** by the resonances of the 2-pyrrolidone ring and the glycerol moiety suggesting them to be diastereoisomers. Due to the lack of sufficient materials, their structural investigation was not completed.

**Scheme 1:**
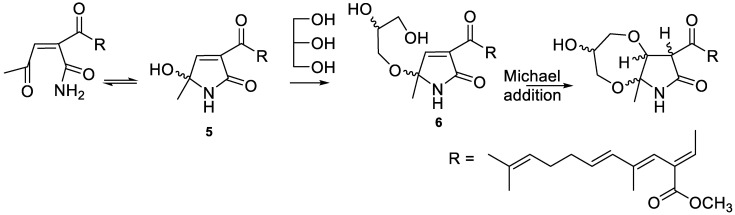
Possible route of formation of the diastereomers of compound **4**.

These diastereomers probably formed from the precursor **6** by Michael addition (Scheme 1) [33]. All the fusarin [33], epolactaene [28], and lucilactaene [34] type compounds so far reported from natural sources have a free hydroxy group at C-15. It is very likely that compound **5** is the precursor of both **3** and **4.** The stereocenter at C-15 undergoes racemization (or epimerization) easily through ring opening, even under mild conditions [33,36]. The formation of methyl or glycerol ethers stabilizes this compound [36], but it is very likely that the product would be racemic in the case of **3** and diastereomeric in the case of **6**. In the chemical synthesis of lucilactaene, the Michael addition occurs spontaneously between the hydroxy group of the hydroxyethyl moiety and Δ^13(14)^ olefinic bond [36]. The glycerol moiety in **6** also has a stereogenic center, and a similar Michael addition with the primary hydroxyl group of the glycerol would create additional stereogenic centers, yielding diastereomers.

**Scheme 2:**
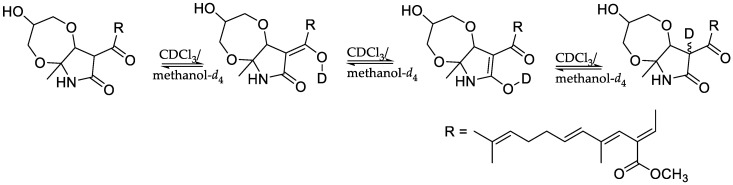
Deuterium exchange of the 2-pyrrolidone ring of compound **4**.

In **4**, the NMR resonance of H-13 was very weak and integrated to less than one proton, and the resonance due to C-13 appeared very small. The 2-pyrrolidone ring in this type of compound could undergo keto-enol tautomerism [36,37], and in deuterated protic solvents it is highly likely that H-13 was partially exchanged with deuterium in CDCl_3_/methanol-*d_4_* in the presence of traces of water or HOD [38] (Scheme 2). The NMR spectra of fusarin A and lucilactaene have previously been recorded in CDCl_3_ [33,34,35,36].

Even though a number of fusarins [33], closely related compounds with fully unsaturated sidechains, have been isolated from different fungi, epolactaene [25] is the only other compound with the same sidechain as those of compounds **3** and **4** that has been reported so far. Unlike our previous studies [8,9,10,11,12] where active metabolites were found in fungi at the stationary phase of growth, which was typically after three weeks, antiplasmodial compound (**4**) in *B. dothidea* appeared in the log phase of growth after about two weeks and disappeared rapidly before the stationary phase of growth. These compounds may be biosynthetic intermediates rather than stable end products. Compounds **3** and **4** are the first natural fusarin type compounds with ether linkages at C-15.

Biological Activity

The 3:1 mixture of compounds **1** and **2** showed strong phytotoxic activity for both representative monocots and dicots (Table 3). Others have reported **1**, **2**, or both compounds to be phytotoxic [22,23,25], using bioassays that did not utilize whole plants. However, they were devoid of antiplasmodial activity (Table 4). The mixture of compound **1** & **2** was evaluated for toxicity against tumor cell lines SK-MEL, KB, BT-549, and SK-OV-3, as well as against the kidney epithelial cell line, LLC-PK11. The mixture showed cytotoxicity towards SK-MEL and SK -OV-3 cancer cell lines and kidney epithelial cells LLC-PK11, but no toxicity against KB and BT-549 cancer cell lines (Table 5). Previously, the enantiomers and racemate of compound **2** have shown moderate antibacterial activity against *Bacillus subtilis* and strong cytotoxic activity against human leukemia HL-60 cells that was comparable to the activity shown by the positive control, irinotecan [39].

Compound **3** was found to be inactive in antiplasmodial assays. The fraction containing compound **4** showed moderate in vitro antiplasmodial activity against chloroquine-sensitive (D6) and -resistant (W2) strains of *P. falciparum* (IC_50_ < 0.523 µg/mL) with no cytotoxicity to Vero cells (Table 4). Lack of sufficient material and instability prevented further biological studies on these compounds.

It is interesting that a related compound with a fully unsaturated sidechain, lucilactaene (Figure 1), has also shown potent antiplasmodial activity [35]. It has been found that the tetrahydropyran ring and methylation of the acid group of the sidechain are essential for the activity of this compound. Chemical instability and various other biological activities reported for this class of compounds [33,34,35,36] would preclude them from being potential antimalarial agents.

## 3. Materials and Methods

### 3.1. General Experimental Procedures

NMR spectra were recorded on a Varian 400 MHz and/or Varian 600 MHz spectrometer (Varian, Palo Alto, CA, USA ) using CDCl_3_ or CDCl_3_/methanol-*d*_4_ (4:1) as the solvent, unless otherwise stated. MS analyses were performed on an Agilent Series 1100 SL equipped with an ESI source (Agilent Technologies, Palo Alto, CA, USA). Column chromatography was carried out on silica gel 60 (230–400 mesh) (Sigma-Aldrich, St. Louis, MO, USA) and Sephadex LH-20 (105 × 3 and 69 × 2 cm^2^) (GE Healthcare Bio-Science, Marlborough, MA, USA). HPLC analysis was carried out on a Hewlett Packard 1100 series instrument with Luna C18 columns (10µ C_18_ 250 × 4.6 mm^2^, 10 micron; 10μ C_18_ 250 × 2 mm^2^, 10 micron; Phenomenex (Torrance, CA, USA) as the stationary phase and methanol-water (1:4) as the mobile phase. TLC spots were detected under UV light and by heating after spraying with anisaldehyde reagent.

### 3.2. Isolation of the Fungus from a Seed of Diseased T. taxifolia

Seeds were collected from a *T. taxifolia* tree with disease symptoms cultivated on the Biltmore Estate in Asheville, North Carolina. A voucher of the *T. taxifolia* Arn. plant was identified by E. M. Croom, Jr. and deposited in the University of Mississippi Pullen Herbarium. The voucher accession number is MISS 55406.

A seed of *T. taxifolia* was surface disinfected by immersing in 70% EtOH (1 min) and 2% NaOCl (3 min), followed by washing with sterile distilled water (2 min). The seed was subsequently fragmented and plated onto Petri dishes containing potato dextrose agar (PDA; BD Difco, Franklin Lakes, NJ, USA) supplemented with 200 mg/L chloramphenicol to avoid bacterial contamination. The plates were incubated at 25 °C for 60 days. Hyphal growth was monitored over an 8-week period. Using an aseptic technique, the endophyte was transferred to PDA contained in 60-mm Petri plates. The long-term preservation of filamentous fungal colonies was carried out in cryotubes containing 15% sterile glycerol at −80 °C.

### 3.3. Identification of the Fungus by DNA Analysis

DNA isolation, PCR amplification, cloning, and sequencing were performed as described in Kumarihamy et al. 2019 [12]. Homology searches were performed with the Basic Local Alignment Search Tool (BLAST) [40]. The UM124 sequence was submitted to GenBank (Accession MK679616).

A phylogenetic tree [12] was constructed to identify close relatives of UM124 with the best 19 hits from BLAST and already published sequences in Genebank of various *Botryosphaeria* species. In addition, a phylogenetic tree of UM124 and sequences from various taxa of the family *Botryosphaeriaceae* was constructed (Supplementary Materials: Tables S1 and S2 and Figures S1 and S2).

### 3.4. Fermentation, Extraction, and Purification

*B. dothidea* was cultured in 80 conical flasks (1 L) containing 500 mL of potato dextrose broth and incubated at 27 °C for 14 days on an orbital shaker at 100 rpm. The mycelium was separated by filtration, and the broth was extracted with an equal amount of EtOAc (×3). The EtOAc extract was evaporated to give a black residue (3.15 g).

A portion of the EtOAc extract (3 g) was chromatographed over silica gel and eluted with a gradient of hexanes, CH_2_Cl_2_ and MeOH to yield 15 fractions. Fractions which showed antimalarial activity were combined (550 mg), chromatographed over Sephadex LH-20 (105 × 3 cm), and eluted with MeOH to give 12 fractions. A white precipitate observed in subfraction 11 was separated and washed with Et_2_O to yield a 3:1 mixture (10 mg) of **1** and **2** as white amorphous powders. Their identity was confirmed by comparing ^1^H and ^13^C NMR, and HRESIMS data with the literature data [22,23,24,25,26].

Subfractions 6–10, which showed antimalarial activity, were combined (60 mg) and further separated using a C_18_ reversed-phase preparative HPLC column (Luna 10µ C_18_ 250 × 10 mm, 10 micron) and was eluted with MeOH-H_2_O (1:4) at a flow rate of 3.0 mL/min to give two major peaks. Peak one, which showed no antimalarial activity, was further purified by Sephadex LH-20 (60 × 2 cm) gel filtration with MeOH (100%) to give a new compound (**3**, 1.5 mg).

Peak 2, which showed moderate antimalarial activity, was purified by using C_18_ reversed-phase analytical HPLC chromatography (Luna 10µ C_18_ 250 × 4.6 mm, 10 micron) and was eluted with MeOH-H_2_O (1:4) at a flow rate of 1.5 mL/min to give **4** (1.0 mg) and two additional components.

Compound **3**: HRESIMS [M + H]^+^ *m/z* 388.2184 (calcd for [C_22_H_29_NO_5_ + H]^+^ 388.2124), ^1^H- and ^13^C-NMR data: see Table 2.

Compound **4**: HRESIMS [M + H]^+^ *m/z* 448.2268 (calcd for [C_24_H_33_NO_7_ + H]^+^ 448.2335), ^1^H- and ^13^C-NMR data: see Table 2.

### 3.5. In Vitro Antiplasmodial Assay

The antiplasmodial assay was performed against D6 (chloroquine sensitive) and W2 (chloroquine resistant) strains of *P. falciparum* using the in vitro assay as reported earlier [41]. Artemisinin and chloroquine were included as the drug controls, and IC_50_ values were computed from the dose-response curves.

### 3.6. In Vitro Phytotoxicity Assay

Herbicidal or phytotoxic activity of the extract and compounds was performed according to a published procedure [42] using bentgrass (*Agrostis stolonifera*) and lettuce (*Lactuca sativa* cv. L., Iceberg), in 24-well plates. Phytotoxicity was ranked visually. The ranking of phytotoxic activity was based on a scale of 0 to 5 with 0 showing no effect and 5 showing no growth.

### 3.7. In Vitro Cytotoxicity Assay

In vitro cytotoxicity was determined against a panel of mammalian cells that included kidney fibroblast (Vero), kidney epithelial (LLC-PK_11_), malignant melanoma (SK-MEL), oral epidermal carcinoma (KB), breast ductal carcinoma (BT-549), and ovarian carcinoma (SK-OV-3) cells [12]. Cells were seeded to the wells of a 96-well plate at a density of 25,000 cells/well and incubated for 24 h. Samples at different concentrations were added and plates were again incubated for 48 h. The number of viable cells was determined by using neutral red dye and IC_50_ values were obtained from dose response curves. Doxorubicin was used as a positive control.

## 4. Conclusions

Bioactivity-guided fractionation of the EtOAc extract of the broth of *B. dothidea* isolated from a seed of a diseased *T. taxifolia* plant afforded a mixture of two known isomeric 2,4-pyridione epoxides, FRT-A (**1**) and flavipucine (**2**) (or their enantiomers, sapinopyridione and (−)-flavipucine) and two new *α*-alkyl-*γ*-lactam alkaloids, dothilactaene A (**3**), and dothilactaene B (**4**). The mixture of 2,4-pyridione epoxides, displayed strong phytotoxicity against both a dicot and a monocot and moderate cytotoxicity against a panel of cell lines but no antiplasmodial activity. Dothilactaene A showed no activity. Dothilactaene B was isolated from the active fraction, which showed moderate in vitro antiplasmodial activity with high selectivity index. In spite of this activity, its instability and various other biological activities shown by related compounds would preclude it from being a viable antimalarial lead.

## Figures and Tables

**Table 1:** ^1^H- and ^13^C-NMR data for mixture of compounds **1** and **2** (3:1) in CDCl_3._

Sapinopyridione [25]			Compound 1		(-)-Flavipucine [22,27]		Compound 2	
C	*δ*_C_	*δ*_H_	*δ*_C_^a^	*δ*_H_^b^ (*J* in Hz)	*δ*_C_	*δ*_H_	*δ*_C_^a^	*δ*_H_^b^ (*J* in Hz)
2	68.6	3.94, s	68.7	3.94, s	68.8	3.86, s	68.8	3.84, s
3	59.9		60.0		60.0		59.8	
4	168.2		168.7		168.8		168.6	
6	154.9		155.8		156.2		155.8	
7	107.1	5.64, s	107.2	5.63, s	107.3	5.64, m	107.2	5.63, s
8	186.2		186.5		186.9		186.4	
9	206.5		206.6		203.6		203.7	
10	44.3	3.28, ddq	44.5	3.22, m	49.6	2.68, m	49.6	2.62, dd (16.7, 7.2) 2.68, dd (16.7, 7.2)
11	25.3	1.79, ddq	25.4	1.78, m	24.1	2.1, m	24.0	2.17 ^c^
		1.52, ddq		1.50, m				
12	11.1	0.97, t	11.2	0.94, t (7.4)	22.5	0.94, d (6.6)	22.6	0.92, d ( 6.7)
13	12.6	1.05, d	12.7	1.02, d (6.6)	22.8	0.98, d (6.6)	22.9	0.96, d, (6.7)
14	20.8	2.19, s	20.8	2.16, s	20.8	2.18, d (1.0)	20.8	2.16, s
NH		8.38, br s		9.24, br s		9.14, br s		9.22, br s

^a^ Recorded at 100 MHz, ^b^ Recorded at 400 MHz, ^c^ Overlapped signals.

**Table 2:** ^1^H- and ^13^C-NMR spectroscopic data for compounds **3** and **4.**

	3 (CDCl_3_)		4 (CDCl_3_:methanol-*d*_4_ 4:1)	
	*δ*_C_^a^	*δ*_H_^b^ (*J* in Hz)	*δ*_C_^a^	*δ*_H_^b^ (*J* in Hz)
1	15.9	1.72, d (7.1)	15.8	1.55, d (7.1)
2	139.9	6.96, q (7.1)	140.1	6.76, q (7.1)
3	130.3		130.4	
4	123.1	5.97, br s	122.7	5.76, s
5	137.7		138.0	
6	135.6	6.23, d (15.6)	135.0	6.10, d (15.6)
7	128.2	5.68, dt (15.2, 6.6)	128.8	5.60, dt (15.4, 6.9)
8	31.5	2.32, m	31.6	2.20, m
9	28.4	2.45, m	29.4	2.30, m
10	148.6	6.56, t (6.4)	146.6	6.59, t (4.6)
11	137.8		138.4	
12	191.4		196.2	
13	139.1		50.0	3.21, s
14	149.5	6.83, s	76.5	4.29, s
15	89.2		83.9	
17	168.3		168.3	
18	24.8	1.61, s	20.3	1.46, s
19	167.8		168.3	
20	51.9	3.74, s	51.9	3.55, s
21	14.3	1.62, s	14.3	1.45, s
22	11.1	1.89, s	11.6	1.67, s
23	50.8	3.18, s	60.7	3.45, 3.49, m
24			70.4	3.65, m
25			61.9	3.39, 3.58, m ^c^

^a^ Recorded at 150 mHz, ^b^ Recorded at 600 mHz, ^c^ Overlapped signals.

**Table 3:** Phytotoxic activity of mixture of compounds 1 and 2 ^a^.

Compound	Lettuce	Bentgrass
EtOAc extract	no growth	no growth
3:1 mixture of **1** and **2**	no growth	no growth

^a^ Concentration = 1 mg/mL.

**Table 4:** Antiplasmodial activity of compounds **3** and **4**.

Compound	Chloroquine-Sensitive (D6)-Clone		Chloroquine-Resistant (W2)-Clone		Cytotoxicity to Vero Cells
	IC_50_ µg/mL (µM)	SI	IC_50_ µg/mL (µM)	SI	IC_50_ µg/mL (µM)
3:1 mixture of **1** and **2**	NA		NA		NC
**3**	NA		NA		NC
Fraction containing **4** ^a^	<0.523 (<1.17)	>9	<0.523 (<1.17)	>9	NC
Chloroquine ^b^	0.016 (0.03)	496.6	0.16 (0.31)	48.1	NC
Artemisinin ^b^	0.0056 (0.02)	845	0.003 (0.01)	1690	NC

^a^ this fraction showed 100% inhibition at the lowest concentration tested (0.523 µg/mL). ^b^ Positive controls, IC_50_ = concentration causing 50% growth inhibition, NA = not active at the highest concentration tested (4.76 µg/mL), NC = no cytotoxic at the highest concentration tested (4.76 µg/mL), S. I. (selectivity index) = IC_50_ for cytotoxicity/IC_50_ for antiplasmodial activity.

**Table 5:** Cytotoxic activity [IC_50_ (µM)] of **1** and **2** mixture.

Compound	SK-MEL	KB	BT-549	SK-OV-3	LLC-PK_11_
3:1 mixture of **1** and **2**	5.05	21.9	20.3	6.7	7.6
Doxorubicin ^a^	1.29	2.12	1.83	1.47	1.28

^a^ Positive control, IC_50_ = concentration causing 50% growth inhibition, SK-MEL = human malignant melanoma, KB = human epidermal carcinoma, BT-549 = human breast carcinoma (ductal), SK-OV-3 = ovarian carcinoma, LLC-PK_11_ = kidney epithelial cells.

## Data Availability

The data presented in this study are available in this article or supplementary material.

## Supplementary Materials

The following are available online. Figure S1: Constructed tree using Neighbor-Joining method using MEGA X software to match UM124 to already published sequences of family *Botryosphaeriaceae* taxa to help identifying close relatives. Figure S2: Constructed tree using Neighbor-Joining method using MEGA X software to match UM124 to already published sequences to help identifying close relatives. Figures S3–S24: NMR spectra and HRMS data of compounds **1**–**4**, Figures S25–S38: NMR spectra and HRMS data of two additional components (compounds **7** and **8**) isolated from the active fraction. Table S1: Best nineteen hits 100% sequence identity, Table S2: ITS sequences of taxa of the *Botryosphaeriaceae* used for alignment. Table S3: ^1^H and ^13^C NMR data for mixture of compounds **1** & **2**.

## Sample Availability

Samples of the compounds **1**–**4** are not available from the authors.

## References

[1] Lim L., McFadden G.I. (2010). The evolution, metabolism, and functions of the apicoplast. Phil. Trans. R. Soc. B.

[2] Arisue N., Hashimoto T. (2015). Phylogeny and evolution of apicoplasts and apicomplexan parasites. Parasitol. Int..

[3] Ralph S.A., van Dooren G.G., Waller R.F., Crawford M.J., Fraunholz M.J., Foth B.J., Tonkin C.J., Roos D.S., McFadden G.I. (2004). Tropical infectious diseases: Metabolic maps and functions of the *Plasmodium falciparum* apicoplast. Nat. Rev. Micro..

[4] Ralph S.A., D’Ombrain M.C., McFadden G.I. (2001). The apicoplast as an antimalarial drug target. Drug Resist. Update..

[5] Botté C.Y., Dubar F., McFadden G.I., Maréchal E., Biot C. (2012). *Plasmodium falciparum* apicoplast drugs: Targets or off-targets?. Chem. Rev..

[6] Uddin T., McFadden G.I., Goodman C.D. (2018). Validation of putative apicoplast-targeting drugs using a chemical supplementation assay in cultured human malaria parasites. Antimicrob. Agents Chemother..

[7] Franck E.D., Duke S. (2014). Natural compounds as next-generation herbicides. Plant Physiol..

[8] Bajsa J., Singh K., Nanayakkara D., Duke S.O., Rimando A.M., Evidente A., Tekwani B.L. (2007). A Survey of synthetic and natural phytotoxic compounds and phytoalexins as potential antimalarial compounds. Biol. Pharm. Bull..

[9] Herath H.M.B.T., Herath W.H.M.W., Carvalho P., Khan S.I., Tekwani B.L., Duke S.O., Tomaso-Peterson M., Nanayakkara N.P.D. (2009). Biologically active tetranorditerpenoids from the fungus *Sclerotinia homoeocarpa* causal agent of dollar spot in turfgrass. J. Nat. Prod..

[10] Kumarihamy M., Fronczek F.R., Ferreira D., Jacob M., Khan S.I., Nanayakkara N.P.D. (2010). Bioactive 1,4-dihydroxy-5-phenyl-2-pyridinone alkaloids from *Septoria pistaciarum*. J. Nat. Prod..

[11] Kumarihamy M., Khan S.I., Jacob M., Tekwani B.L., Duke S.O., Ferreira D., Nanayakkara N.P.D. (2012). Antiprotozoal and antimicrobial compounds from *Septoria pistaciarum*. J. Nat. Prod..

[12] Kumarihamy M., Ferreira D., Croom E.M., Sahu R., Tekwani B.L., Duke S.O., Khan S., Techen N., Nanayakkara N.P.D. (2019). Antiplasmodial and cytotoxic cytochalasins from an endophytic fungus, *Nemania* sp. UM10M, isolated from a diseased *Torreya taxifolia* leaf. Molecules.

[13] Schwartz M.W., Hermann S.M. (1993). The continuing population decline of *Torreya taxifolia* Arn. Bull. Torrey Bot. Club.

[14] Schwartz M.W., Hermann S.M., Vogel C. (1995). The catastrophic loss of *Torreya taxifolia*: Assessing environmental induction of disease hypotheses. Ecol. Appl..

[15] Schwartz M.W., Hermann S.M., van Mantgem P.J. (2000). Estimating the magnitude of decline of the Florida torreya (*Torreya taxifolia* Arn.). Biol. Conserve..

[16] Lee J.C., Yang X., Schwartz M., Strobel G., Clardy J. (1995). The relationship between an endangered North American tree and an endophytic fungus. Chem. Biol..

[17] Smith J.A., O’Donnell K., Mount L.L., Shin K., Detemann R. (2011). A novel *Fusarium* species causes a canker disease of the critically endangered conifer, *Torreya taxifolia*. Plant Dis..

[18] Aoki T., Smith J.A., Mount L.L., Geiser D.M., O’Donnell K. (2013). *Fusarium torreyae* sp. nov., a pathogen causing canker disease of Florida torreya (*Torreya taxifolia*), a critically endangered conifer restricted to northern Florida and southwestern Georgia. Mycologia.

[19] Yang T., Groenewald J.Z., Cheewangkoon R., Jami F., Abdollahzadeh J., Lombard L., Crous P.W. (2017). Families, genera, and species of Botryosphaeriales. Fungal Biol..

[20] Marsberg A., Kemler M., Jami F., Nagel J.H., Postma-Smidt A., Naidoo S., Wingfield M.J., Crous P.W., Spatafora J.W., Hesse C.N. (2017). *Botryosphaeria dothidea*: A latent pathogen of global importance to woody plant health. Mol. Plant Pathol..

[21] Xiao J., Zhang Q., Gao Y.Q., Tang J.J., Zhang A.L., Gao J.M. (2014). Secondary metabolites from the endophytic *Botryosphaeria dothidea* of *Melia azedarach* and their antifungal, antibacterial, antioxidant, and cytotoxic activities. J. Agric. Food Chem..

[22] Sassa T., Onuma Y. (1983). Isolation and identification of fruit rot toxins from the fungus caused *Macrphoma* fruit rot of apple. Agric. Biol. Chem..

[23] Sassa T., Uchie K., Kato H., Onuma Y. (1987). Decomposition of fruit rot toxin A, a host-selective phytotoxin from *Botryosphaeria berengeriana*. Agric. Biol. Chem..

[24] Loesgen S., Bruhn T., Meindl K., Dix I., Schulz B., Zeeck A., Bringmann G. (2011). (+)-Flavipucine, the missing member of the pyridione epoxide family of fungal antibiotics. Eur. J. Org. Chem..

[25] Evidente A., Fiore M., Bruno G., Sparapano L., Motta A. (2006). Chemical and biological characterisation of sapinopyridione, a phytotoxic 3,3,6-trisubstituted-2,4-pyridione produced by *Sphaeropsis sapinea*, a toxigenic pathogen of native and exotic conifers, and its derivatives. Phytochemistry.

[26] Grandolini G., Casinovi C.G., Radics L. (1987). On the biosynthesis of flavipucine. J. Antibiot..

[27] Findlay J.A., Krepinsky J., Shum A., Casinovi C.G., Radics L. (1977). The structure of isoflavipucine. Can. J. Chem..

[28] Kakeya H., Takahashi I., Okada G., Isono K., Osada H. (1995). Epolactaene, a novel neuritogenic compound in human neuroblastoma cells, produced by a marine fungus. J. Antibiot..

[29] Weber D., Gorzalczany S., Martino V., Acevedo C., Sterner O., Anke T. (2005). Metabolites from endophytes of the medicinal plant *Erythrina crista-galli*. Z. Naturforsch. C J. Biosci..

[30] Gao S.S., Li X.M., Du F.Y., Li C.S., Proksch P., Wang B.G. (2010). Secondary metabolites from a marine-derived endophytic fungus *Penicillium chrysogenum* QEN-24S. Mar. Drugs.

[31] Yanga X., Liu Y., Li S., Yang F., Zhao L., Peng L., Ding Z. (2014). Antimicrobial metabolites from endophytic *Streptomyces sp*. YIM61470. Nat. Prod. Commun..

[32] Chagas F.O., Caraballo-Rodríguez A.M., Dorrestein P.C., Pupo M.T. (2017). Expanding the chemical repertoire of the endophyte *Streptomyces albospinus* RLe7 reveals amphotericin B as an inducer of a fungal phenotype. J. Nat. Prod..

[33] Kleigrewe K., Aydin F., Hogrefe K., Piecuch P., Bergander K., Würthwein E.U., Humpf H.U. (2012). Structure elucidation of new fusarins revealing insights in the rearrangement mechanisms of the *Fusarium* mycotoxin fusarin C. J. Agric. Food Chem..

[34] Kakeya H., Kageyama S., Nie L., Onose R., Okada G., Beppu T., Norbury C.J., Osada H. (2001). Lucilactaene, a new cell cycle inhibitor in p53-transfected cancer cells, produced by a *Fusarium* sp.. J Antibiot..

[35] Kato S., Motoyama T., Futamura Y., Uramoto M., Nogawa T., Hayashi T., Hirota H., Tanaka A., Takahashi-Ando N., Kamakura T. (2020). Biosynthetic gene cluster identification and biological activity of lucilactaene from *Fusarium sp*. RK97-94. Biosci. Biotechnol. Biochem..

[36] Yamaguchi J., Kakeya H., Uno T., Shoji M., Osada H., Hayashi Y. (2005). Determination by asymmetric total synthesis of the absolute configuration of lucilactaene, a cell-cycle inhibitor in p53-transfected cancer cells. Angewandte Chemie Int. Ed. Engl..

[37] Lin Y., Wang L., Wang Y., Wang W., Hao J., Zhu W. (2015). Bioactive natural products of *Aspergillus sp*. OUCMDZ-1914, an aciduric fungus from the mangrove soils. Chin. J. Org. Chem..

[38] Nichols M.A., Waner M.J. (2010). Kinetic and mechanistic studies of the deuterium exchange in classical keto-enol tautomeric equilibrium reactions. J. Chem. Educ..

[39] Kusakabe Y., Mizutani S., Kamo S., Yoshimoto T., Tomoshige S., Kawasaki T., Takasawa R., Tsubaki K., Kuramochi K. (2019). Synthesis, antibacterial and cytotoxic evaluation of flavipucine and its derivatives. Bioorg. Med. Chem. Lett..

[40] Altschul S.F., Gish W., Myers E.W., Lipman D.J. (1990). Basic local alignment search tool. J. Mol. Biol..

[41] Bharate S.B., Khan S.I., Yunus N.A., Chauthe S.K., Jacob M.R., Tekwani B.L., Khan I.A., Singh I.P. (2007). Antiprotozoal and antimicrobial activities of *O*-alkylated and formylated acylphloroglucinols. Bioorg. Med. Chem..

[42] Dayan F.E., Romagni J.G., Duke S.O. (2000). Investigating the mode of action of natural phytotoxins. J. Chem. Ecol..

