# Supplementary material for: Antimalarials and Phytotoxins from Botryosphaeria dothidea Identified from a Seed of Diseased Torreya taxifolia
Source: Molecules. 2020 Dec 24;26(1):59. doi: 10.3390/molecules26010059 (PMC7795089; doi:10.3390/molecules26010059)
Supplement: Supplementary file 1 [file molecules-26-00059-s001.pdf]

Supplementary Information

Antimalarials and Phytotoxins from *Botryosphaeria dothidea* identified from a Seed of Diseased *Torreya taxifolia*

Mallika Kumarihamy [1,2,*], Luiz H. Rosa [3], Natascha Techen [1], Daneel Ferreira [2], Edward M. Croom, Jr. [2], Stephen O. Duke [4,†], Babu L. Tekwani [1,‡], Shabana Khan [1,2] and N. P. Dhammika Nanayakkara [1,*]

[1] National Center for Natural Products Research, Research Institute of Pharmaceutical Sciences, School of Pharmacy, The University of Mississippi,University, MS 38677, USA; ntechen@olemiss.edu (N.T.); btekwani@southernresearch.org (B.L.T.); skhan@olemiss.edu (S.K.)
[2] Division of Pharmacognosy, Department of BioMolecular Sciences, School of Pharmacy, The University of Mississippi, University, MS 38677, USA; dferreir@olemiss.edu (D.F.); emcroom@olemiss.edu (E.M.C.J.)
[3] Departamento de Microbiologia, Universidade Federal de Minas Gerais, Belo Horizonte, MG, Brazil; lhrosa@icb.ufmg.br
[4] Natural Products Utilization Research Unit, USDA-ARS, University, MS 38677, USA; sduke@olemiss.edu
* Correspondance: mkumarih@olemiss.edu (M.K.); dhammika@olemiss.edu (N.P.D.N.); Tel.: +1-662-915-1661 (M.K.); +1-662-915-1019 (N.P.D.N.)
† Current address: National Center for Natural Products Research, Research Institute of Pharmaceutical Sciences, School of Pharmacy, The University of Mississippi, University, MS 38677, USA
‡ Current address: Department of Infectious Diseases, Division of Drug Discovery, Southern Research, Birmingham, AL 35205, USA

Contents	Figure	Page #
Constructed tree using Neighbor-Joining method	S1	4
Constructed tree using Neighbor-Joining method	S2	5
^{1}H NMR Spectrum of compound **1** & **2**	S3	6
^{13}C NMR Spectrum of compound **1** & **2**	S4	6
DEPT Spectrum of compound **1** & **2**	S5	7
COSY Spectrum of compound **1** & **2**	S6	7
HMQC Spectrum of compound **1** & **2**	S7	8
HMBC Spectrum of compound **1** & **2**	S8	8
HRMS Data of Compounds **1** & **2**	S9	9
^{1}H NMR Spectrum of compound **3**	S10	10
^{13}C NMR Spectrum of compound **3**	S11	10
DEPT Spectrum of compound **3**	S12	11

COSY Spectrum of compound **3**	S13	11
HMQC Spectrum of compound **3**	S14	12
HMBC Spectrum of compound **3**	S15	12
HRMS Data of Compounds **3**	S16	13
^{1}H NMR Spectrum of compound **4**	S17	13
^{13}C NMR Spectrum of compound **4**	S18	14
DEPT Spectrum of compound **4**	S19	14
COSY Spectrum of compound **4**	S20	15
ROESY Spectrum of compound **4**	S21	15
HMQC Spectrum of compound **4**	S22	16
HMBC Spectrum of compound **4**	S23	16
HRMS Data of Compounds **4**	S24	17
^{1}H NMR Spectrum of compound **7**	S25	17
^{13}C NMR Spectrum of compound **7**	S26	18
DEPT Spectrum of compound **7**	S27	18
COSY Spectrum of compound **7**	S28	19
HMQC Spectrum of compound **7**	S29	19
HMBC Spectrum of compound **7**	S30	20
HRMS Data of Compounds **7**	S31	20
^{1}H NMR Spectrum of compound **8**	S32	21
^{13}C NMR Spectrum of compound **8**	S33	21
DEPT Spectrum of compound **8**	S34	22
COSY Spectrum of compound **8**	S35	22
HMQC Spectrum of compound **8**	S36	23
HMBC Spectrum of compound **8**	S37	23
HRMS Data of Compounds **8**	S38	24

Note; **7** and **8** are two additional components isolated from the active fraction.

**Contents**	**Table**	**Page #**
Best nineteen hits 100% sequence identity	**S1**	25
ITS sequences of taxa of the *Botryosphaeriaceae* used for alignment	**S2**	26 - 28

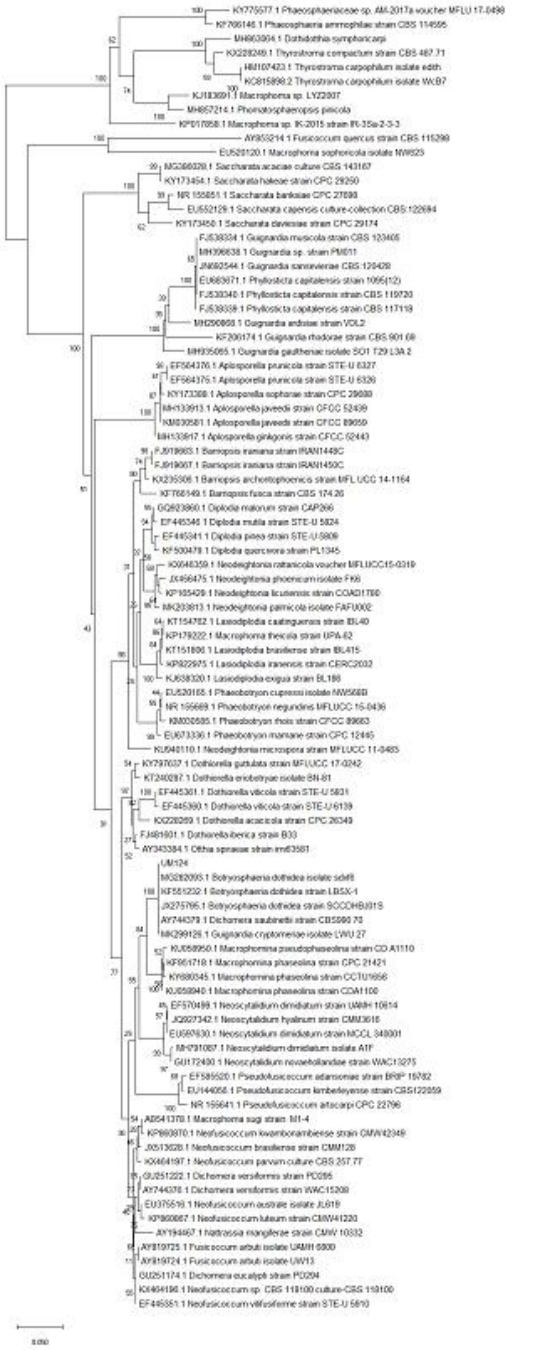

Figure S1: Constructed tree using Neighbor-Joining method using MEGA X software to match UM124 to already published sequences of family *Botryosphaeriaceae* taxa to help identifying close relatives. Numbers displayed on branches are the percentage of replicate trees in which the associated taxa clustered together in the bootstrap test obtained through 500 replications. Sequences used are shown with GenBank accession numbers. UM124 is closely related to *Botryosphaeria dothidea* strains.

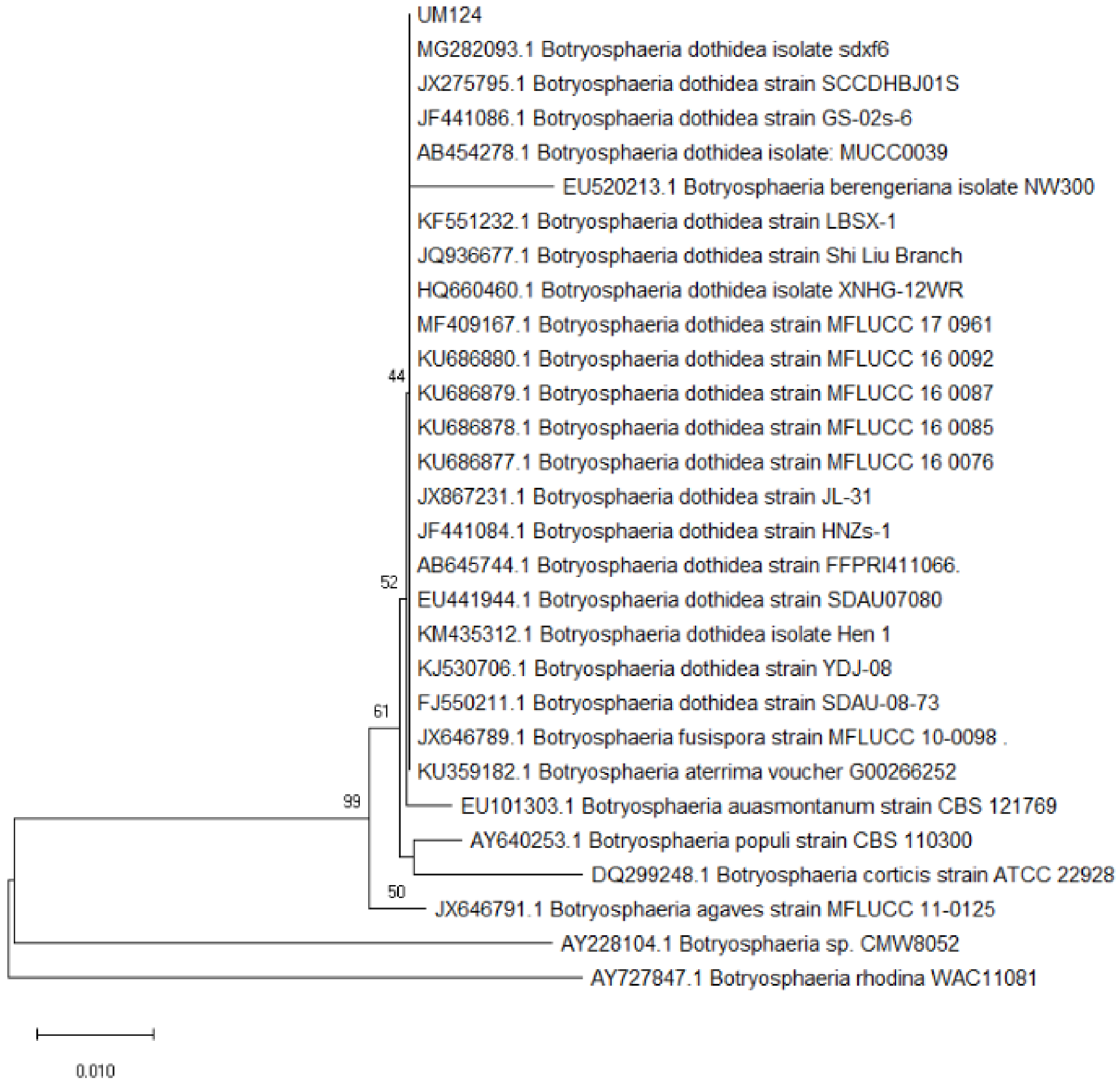

Figure S2: Constructed tree using Neighbor-Joining method using MEGA X software to match UM124 to already published sequences to help identifying close relatives. Numbers displayed on branches are the percentage of replicate trees in which the associated taxa clustered together in the bootstrap test obtained

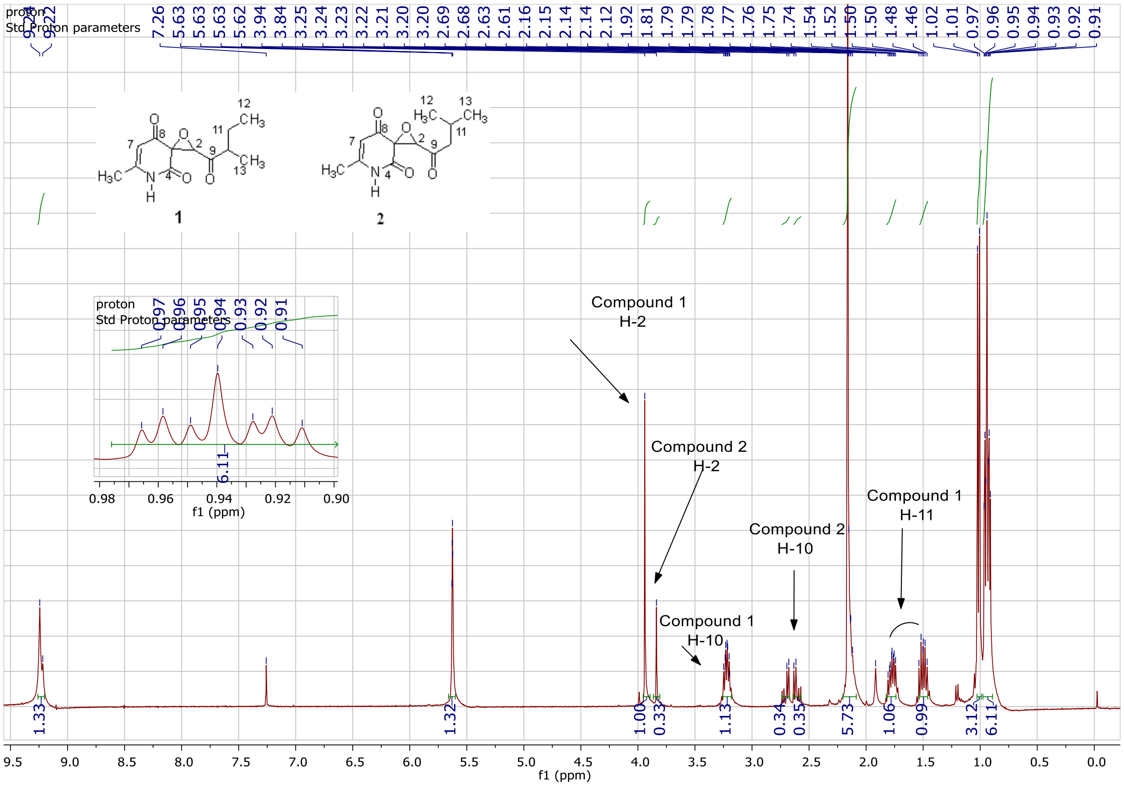

Figure S 3: ^{1}H NMR Spectrum of compounds **1 & 2** in $CDCl_3$

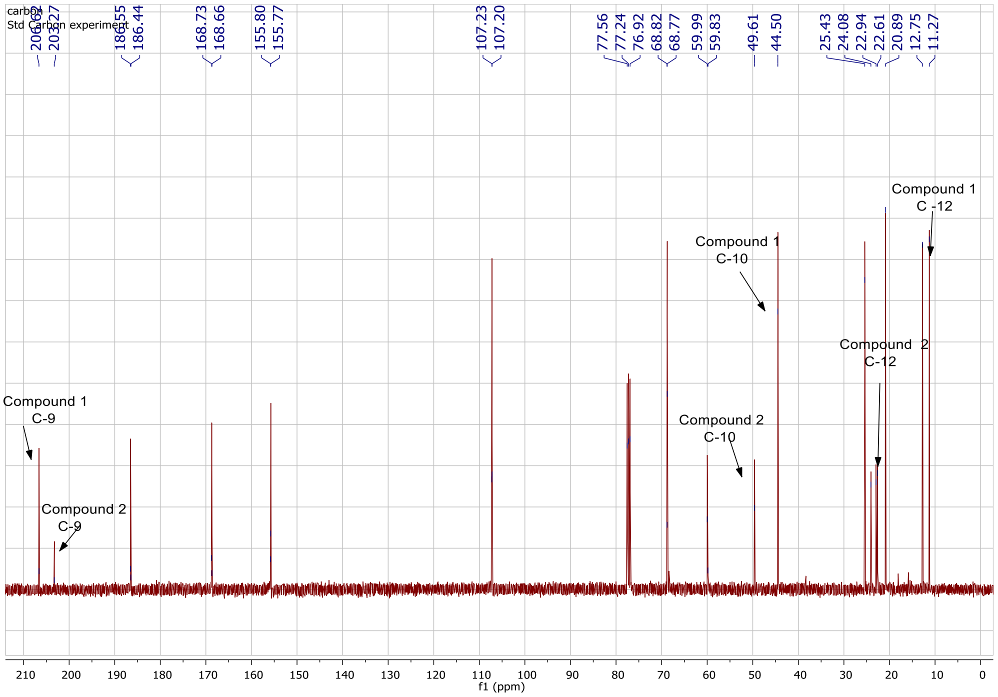

Figure S 4: ^{13}C NMR Spectrum of compounds **1 & 2** in $CDCl_3$

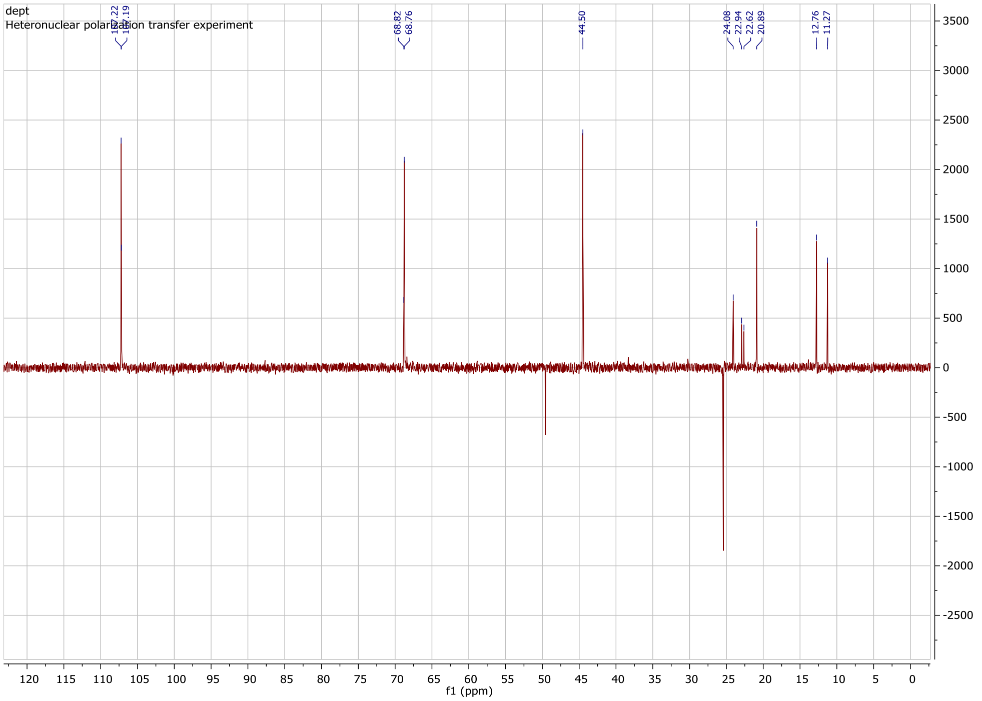

Figure S 5: DEPT Spectrum of compounds **1 & 2** in $CDCl_3$

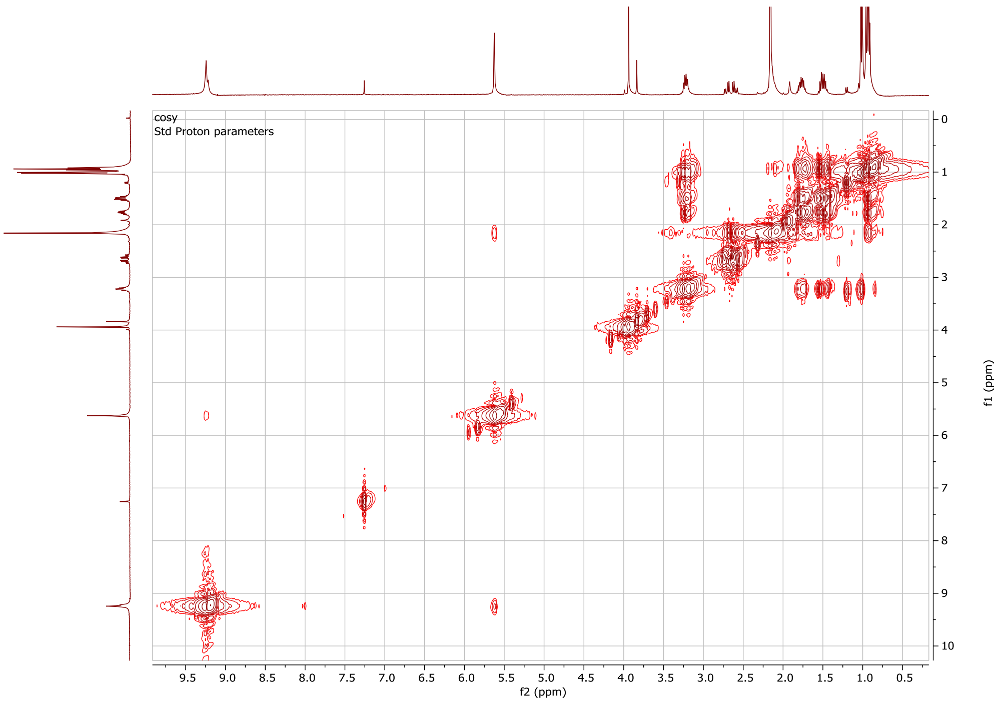

Figure S 6: COSY Spectrum of compounds **1 & 2** in $CDCl_3$

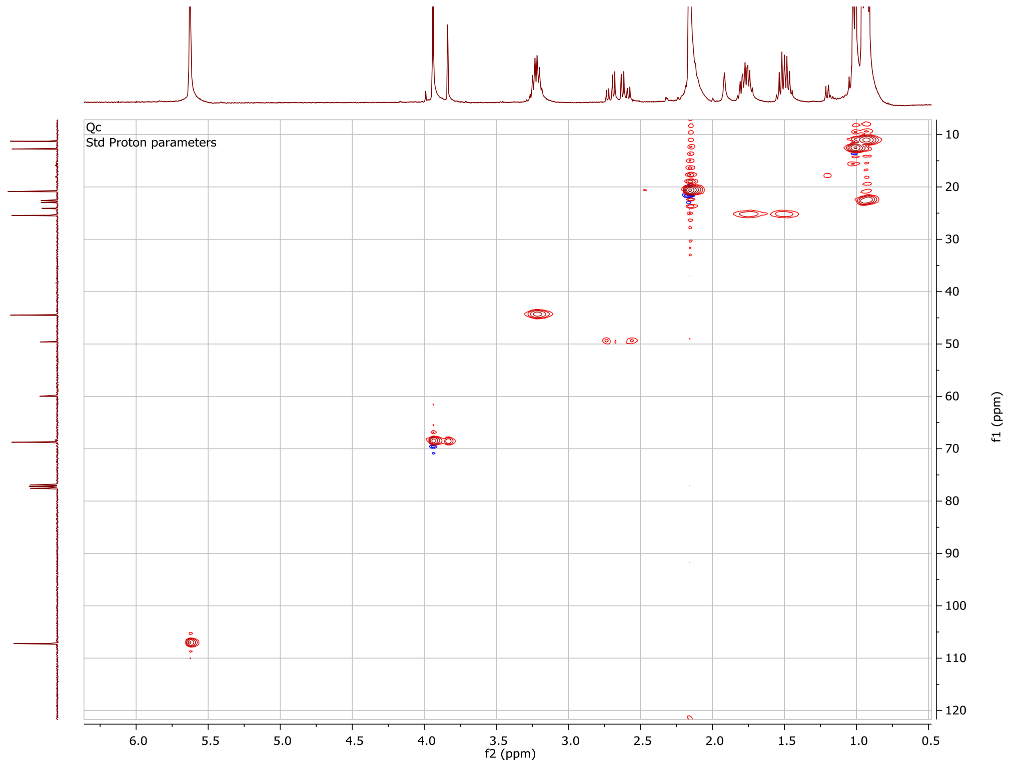

Figure S 7: HMQC Spectrum of compounds **1 & 2** in $CDCl_3$

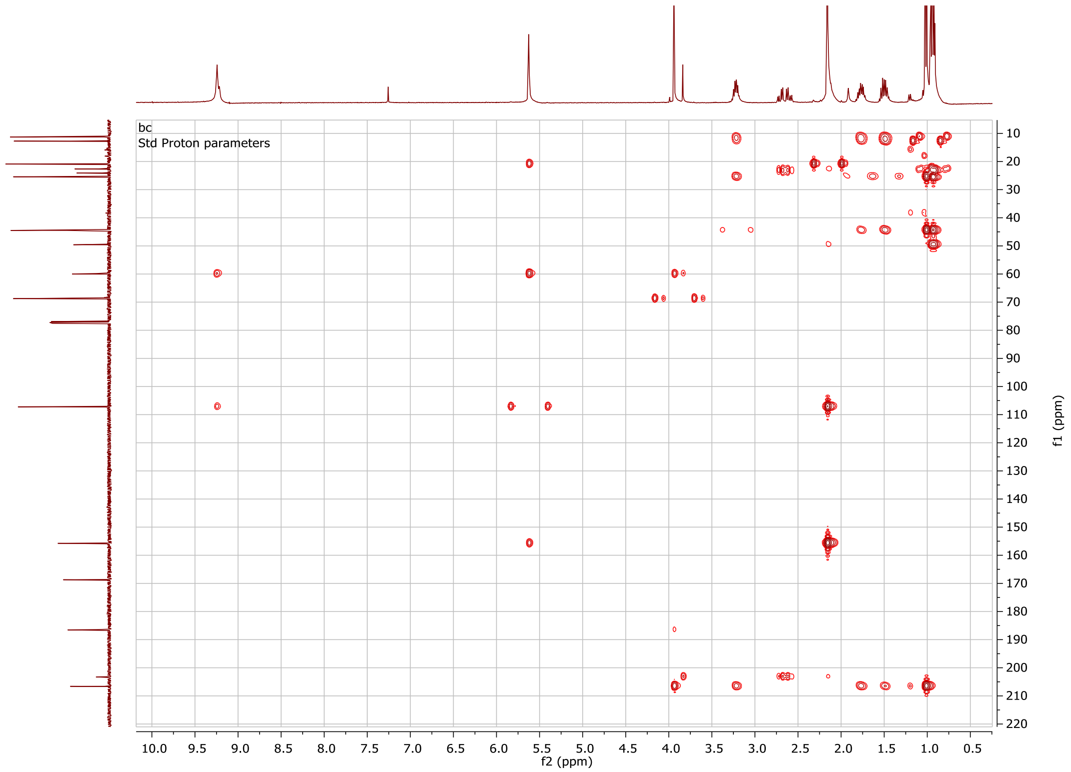

Figure S 8: HMBC Spectrum of compounds **1 & 2** in $CDCl_3$

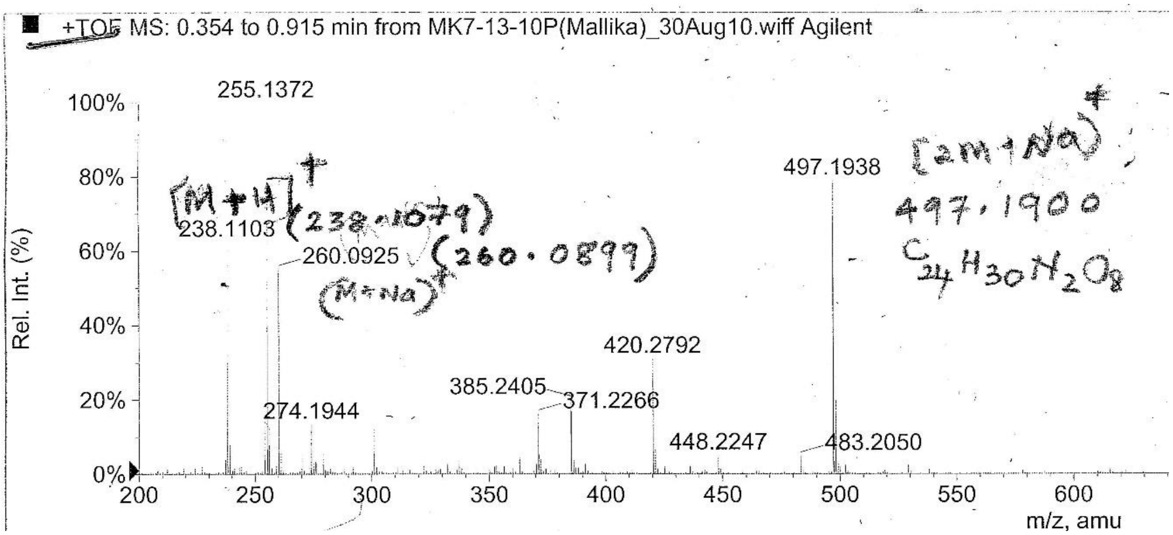

Figure S 9: HR-MS Spectrum of compounds **1 & 2**

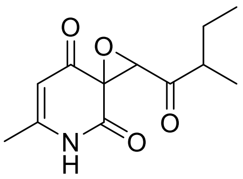

1

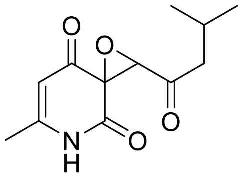

2

M+H

Chemical Formula: $C_{12}H_{16}NO_4$
Exact Mass: 238.1079

M+Na

Chemical Formula: $C_{12}H_{15}NNaO_4$
Exact Mass: 260.0899

2M+Na

Chemical Formula: $C_{24}H_{30}N_2NaO_8$
Exact Mass: 497.1900

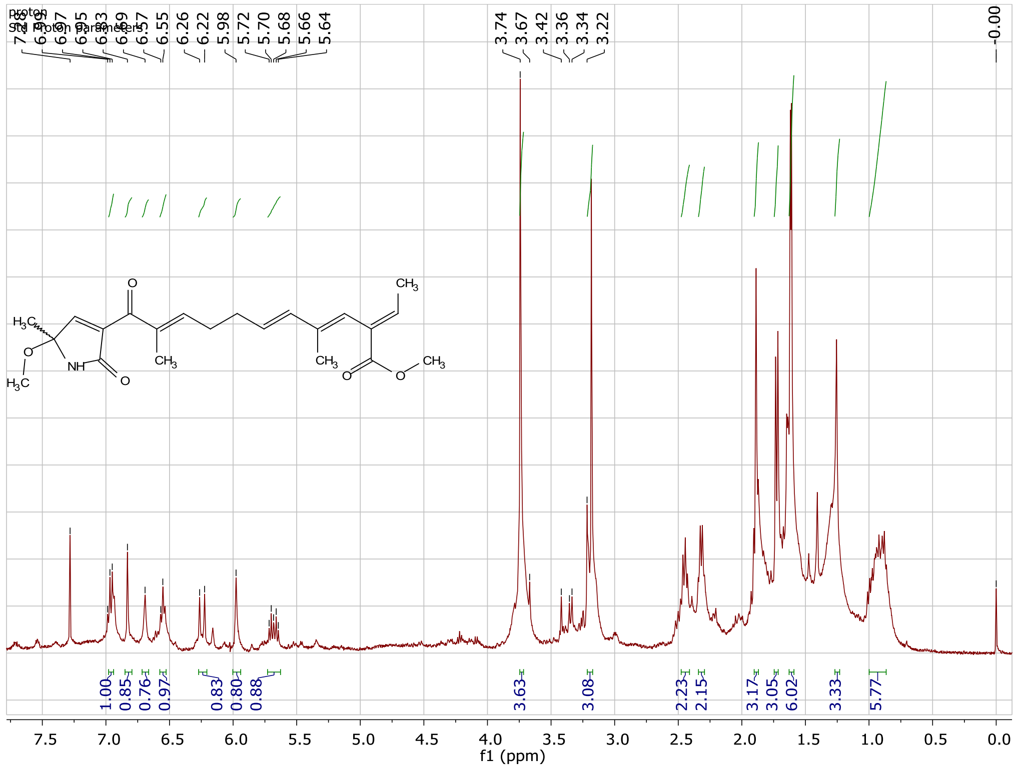

Figure S 10: ^{1}H NMR Spectrum of compound **3** in $CDCl_3$

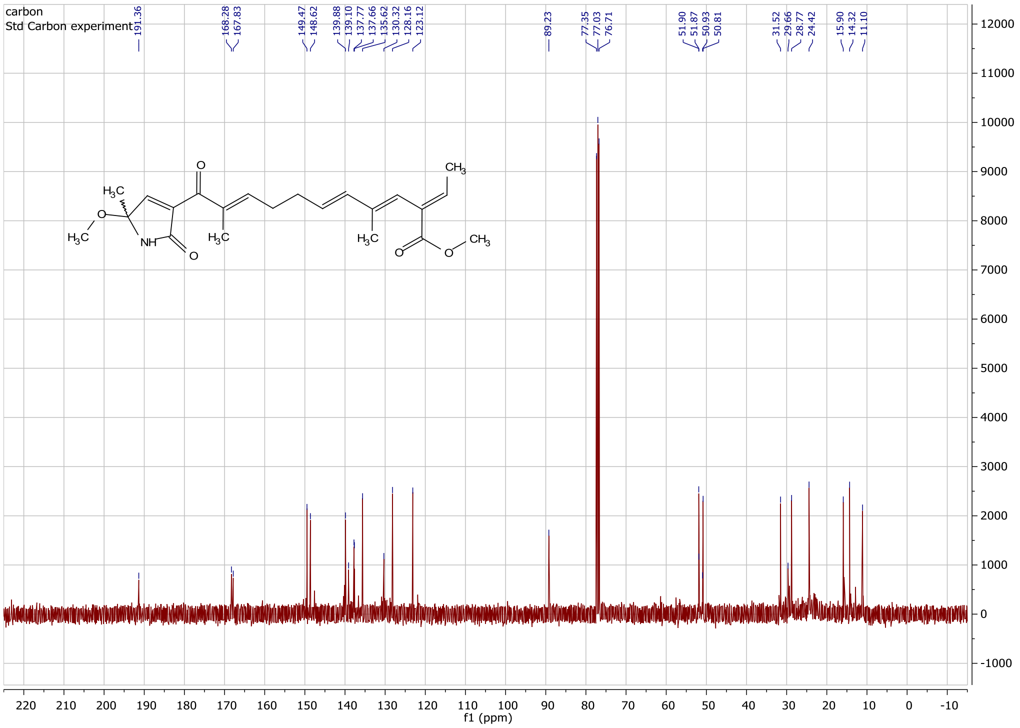

Figure S 11: ^{13}C NMR Spectrum of compound **3** in $CDCl_3$

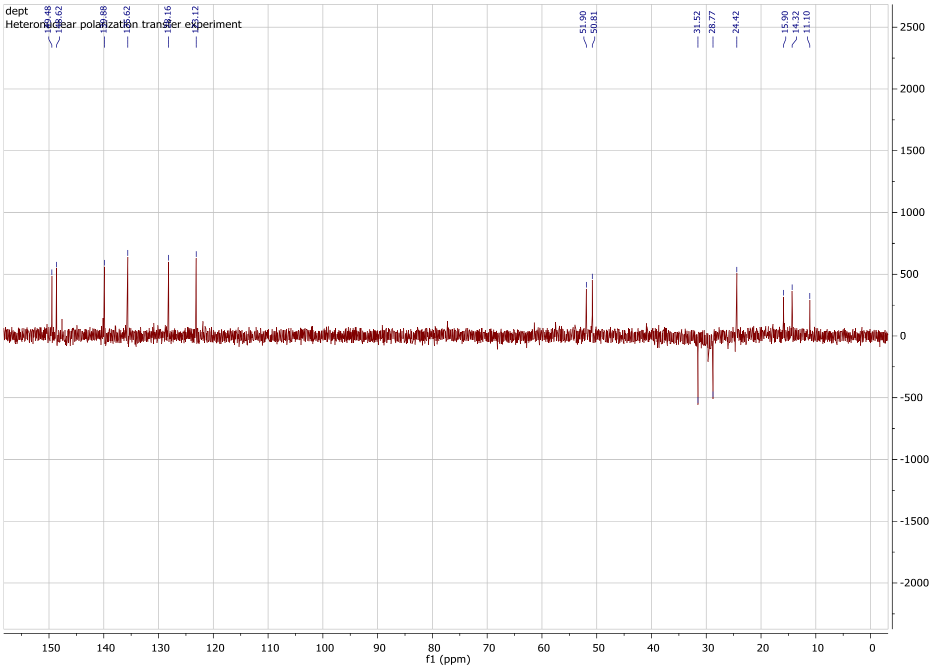

Figure S 12: ^{13}C NMR Spectrum of compound **3** in $CDCl_3$

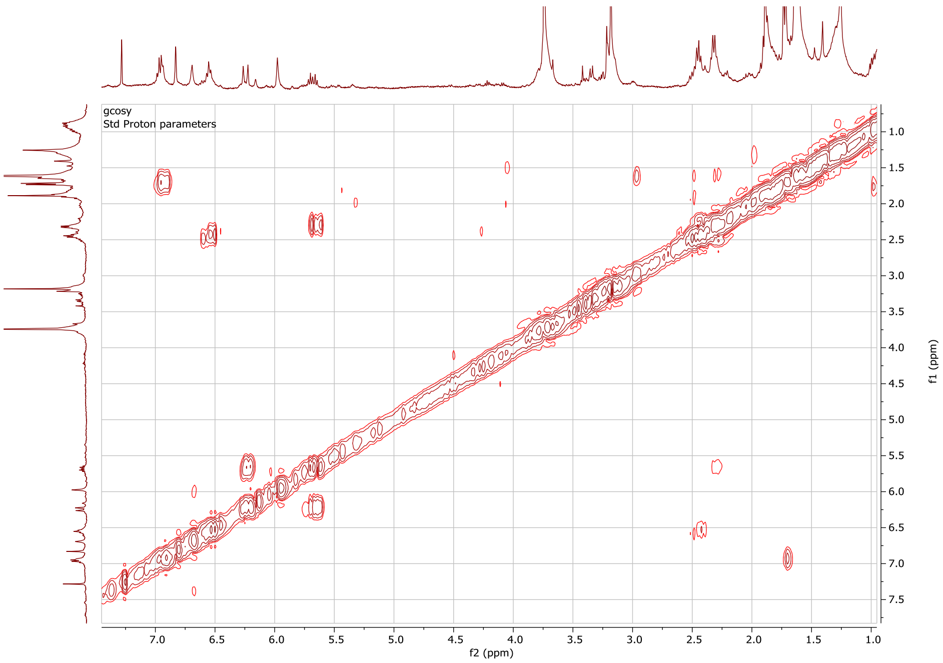

Figure S 13: COSY Spectrum of compound **3** in $CDCl_3$

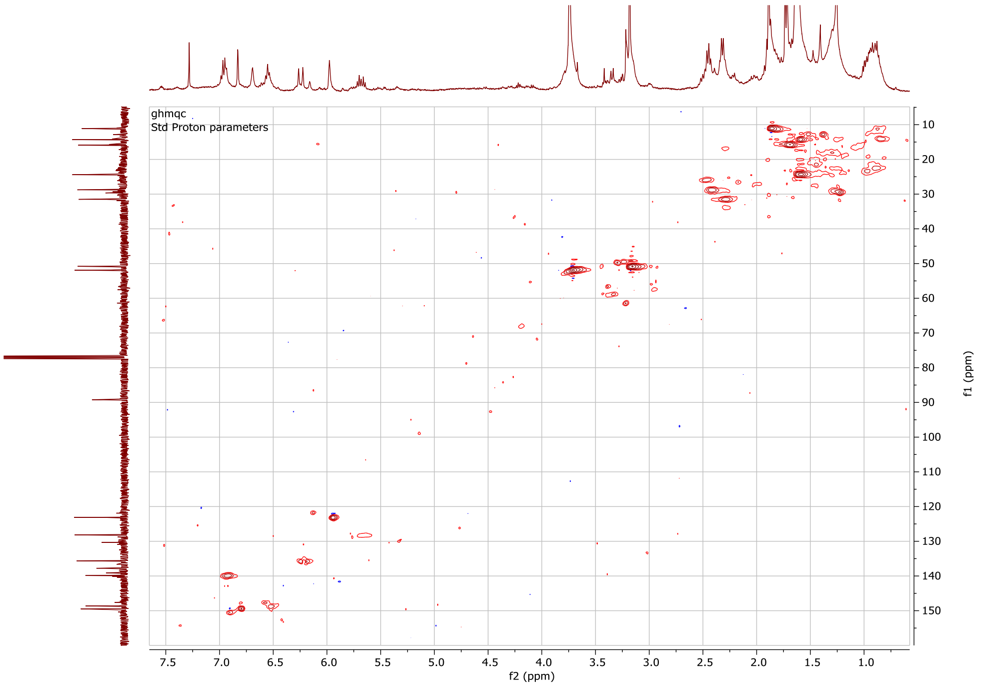

Figure S 14: HMQC Spectrum of compound **3** in $CDCl_3$

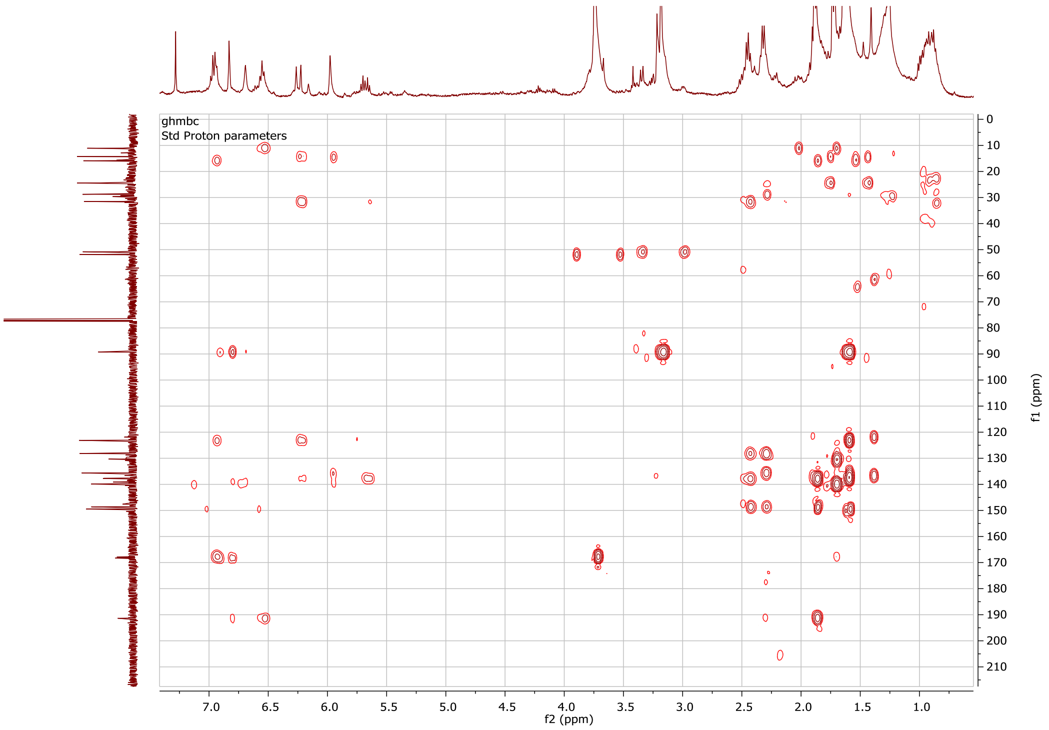

Figure S 15: HMBC Spectrum of compound **3** in $CDCl_3$

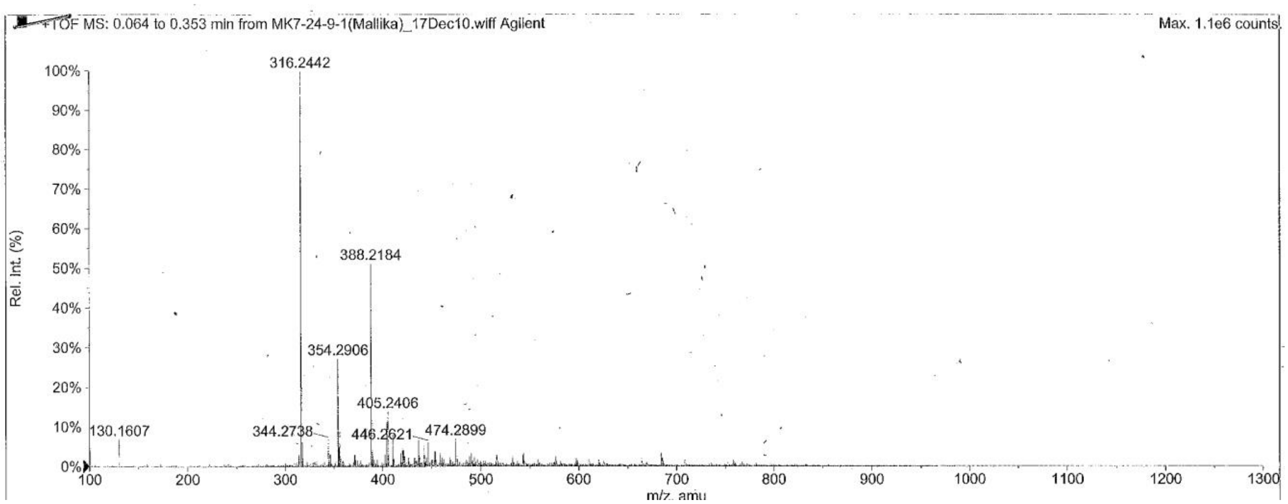

Figure S 16: HRMS Data of Compounds **3**

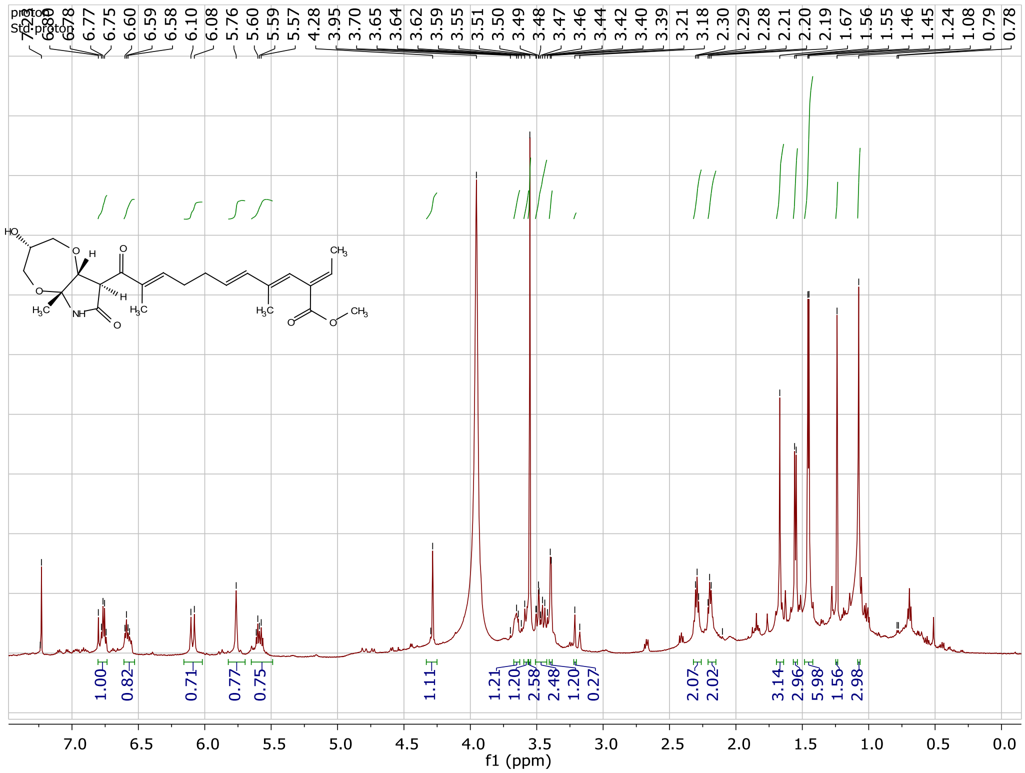

Figure S 17: ^{1}H NMR Spectrum of compound **4** in $CDCl_3$/methanol-d_4 (4:1)

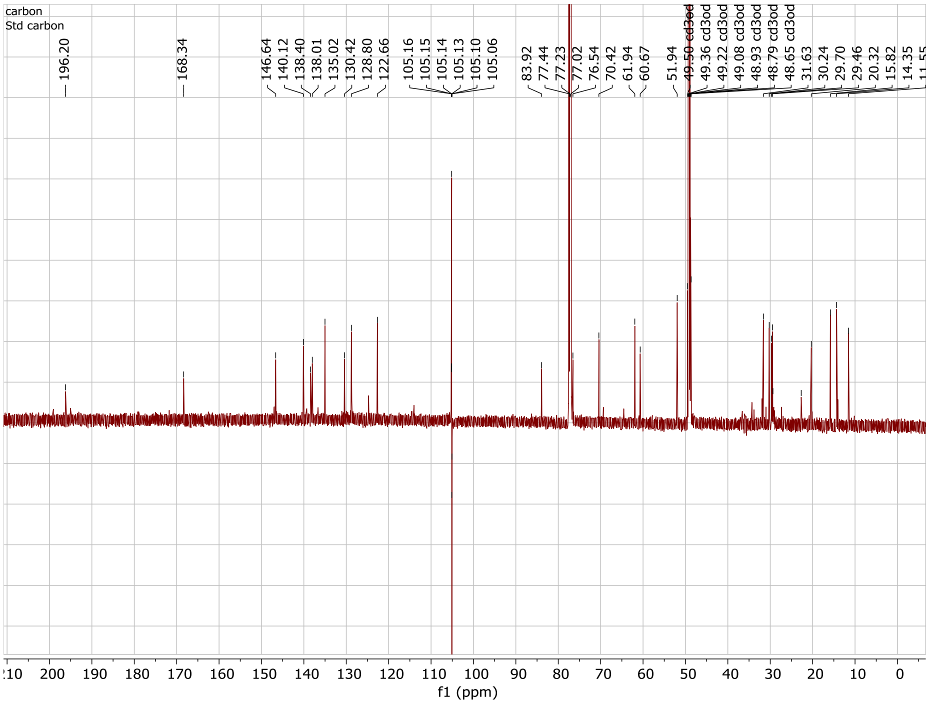

Figure S 18: ^{13}C NMR Spectrum of compound **4** in $CDCl_3$/methanol-d_4 (4:1)

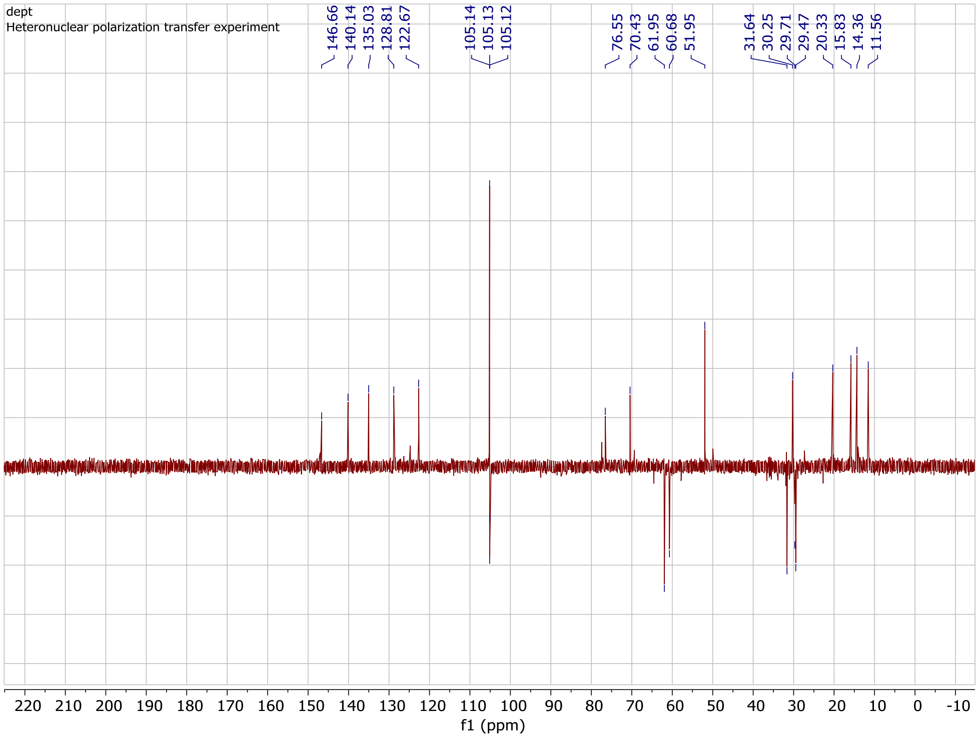

Figure S 19: DEPT Spectrum of compound **4** in $CDCl_3$/methanol-d_4 (4:1)

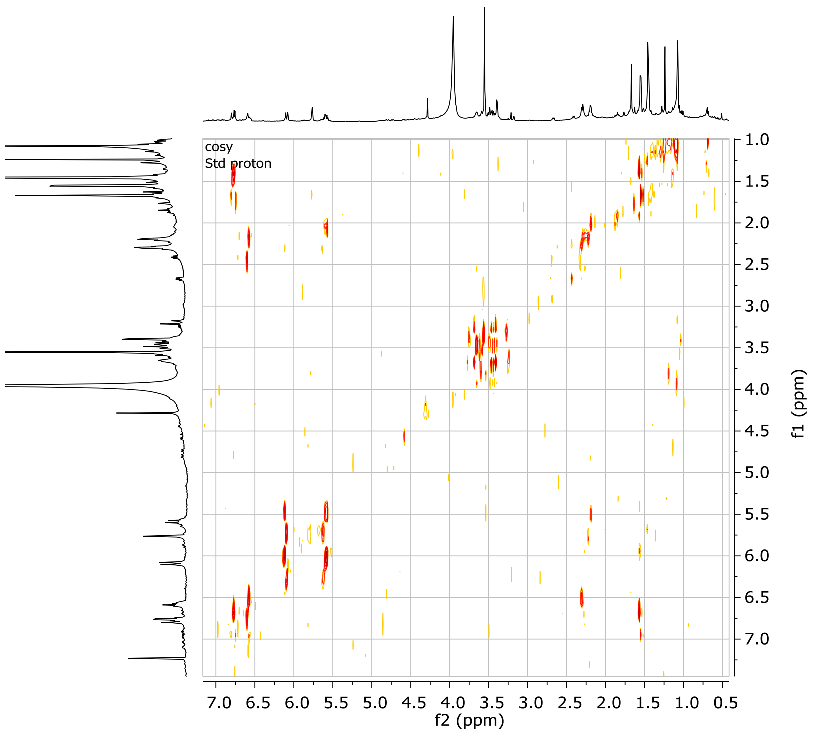

Figure S 20: COSY Spectrum of compound **4** in $CDCl_3$/methanol-d_4 (4:1)

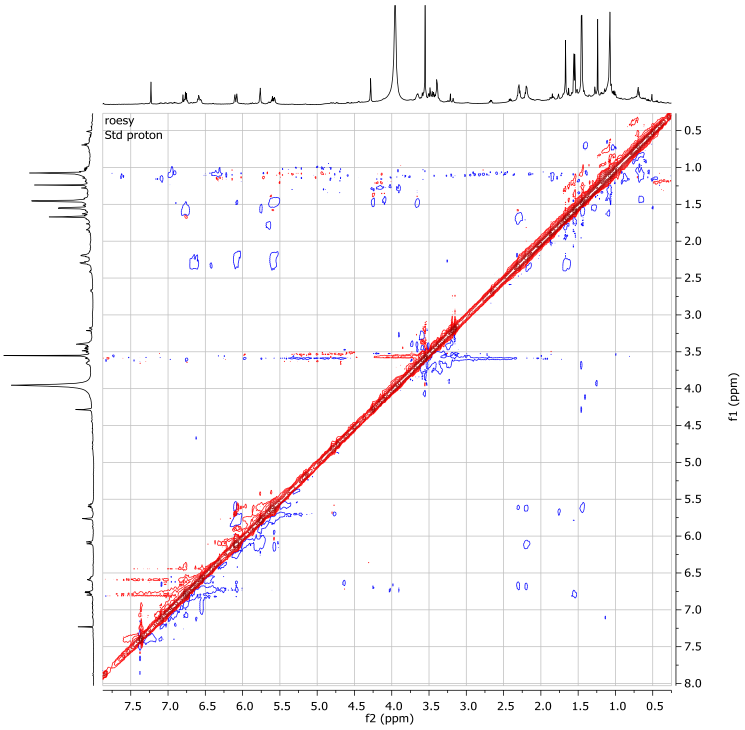

Figure S 21: ROESY Spectrum of compound **4** in $CDCl_3$/methanol-d_4 (4:1)

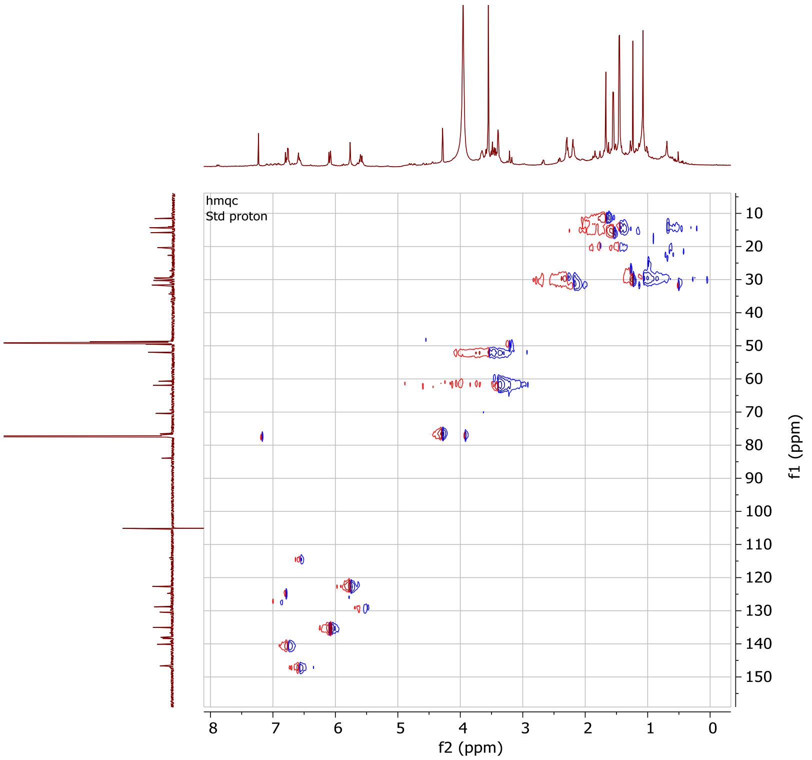

Figure S 22: HMQC Spectrum of compound **4** in $CDCl_3$/methanol-d_4 (4:1)

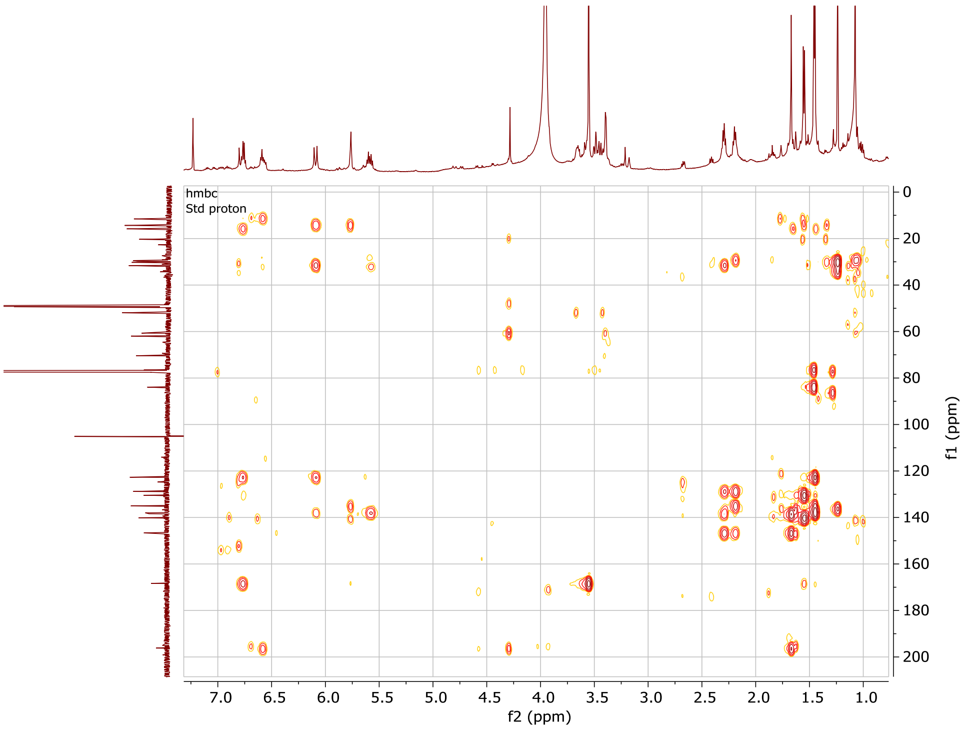

Figure S 23: HMBC Spectrum of compound **4** in $CDCl_3$/methanol-d_4 (4:1)

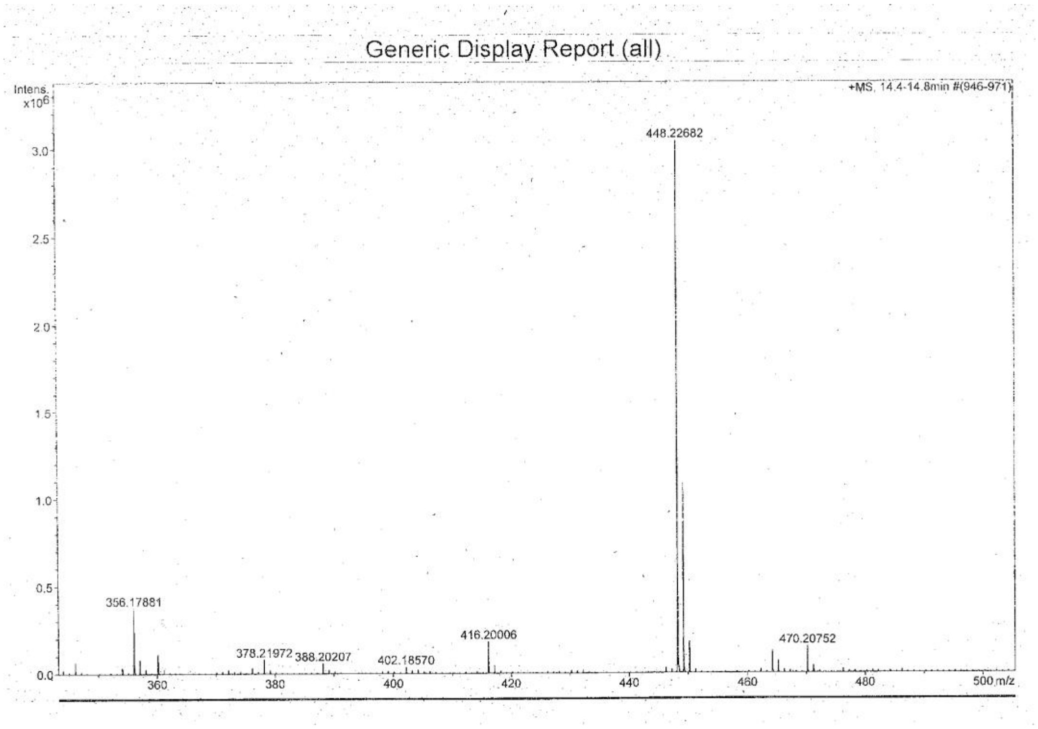

Figure S 24: HRMS Data of Compound **4**

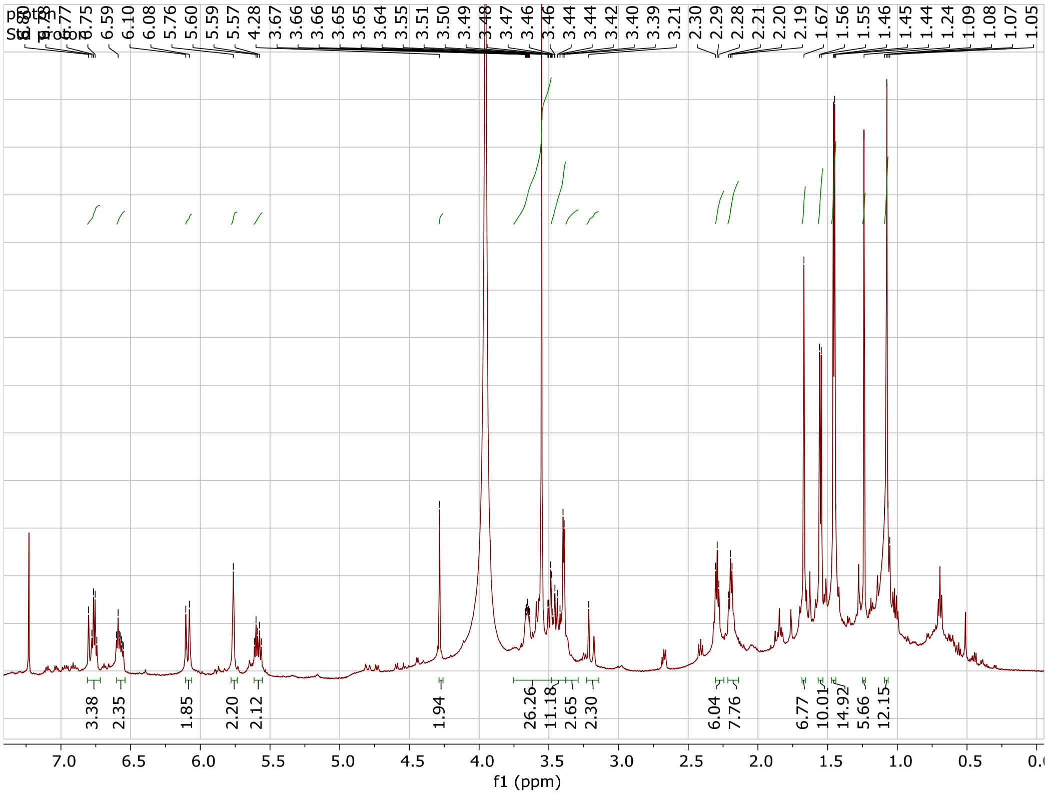

Figure S 25: ^{1}H NMR Spectrum of compound **7** in $CDCl_3$/methanol-d_4 (4:1)

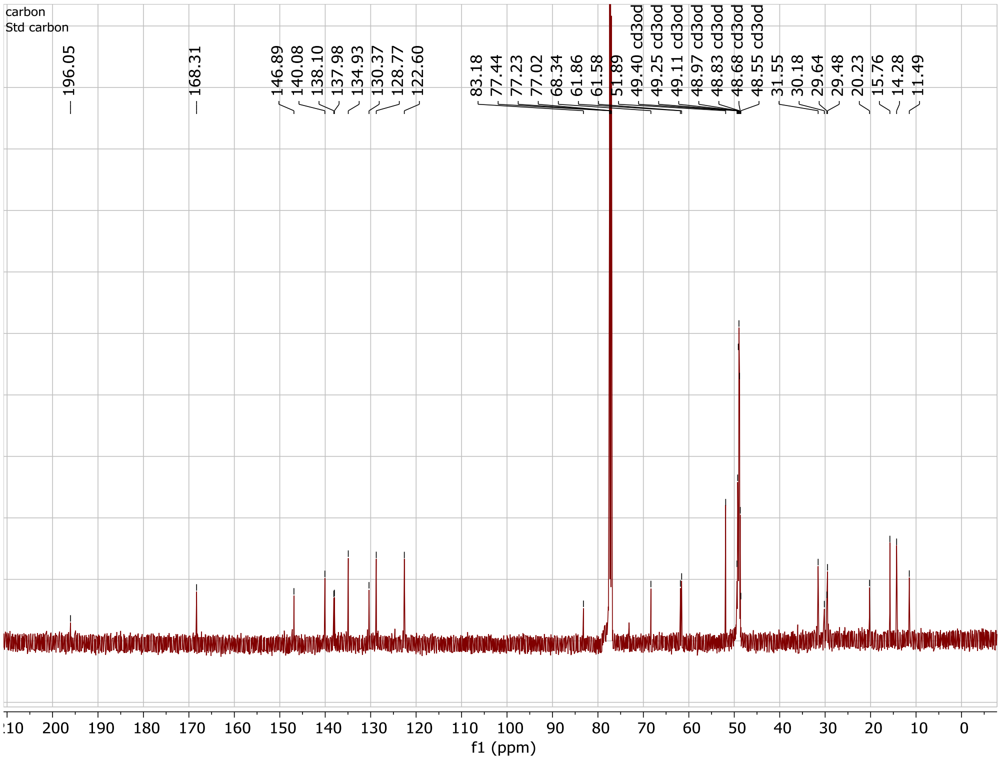

Figure S 26: ^{13}C NMR Spectrum of compound **7** in $CDCl_3$/methanol-*d*$_4$ (4:1)

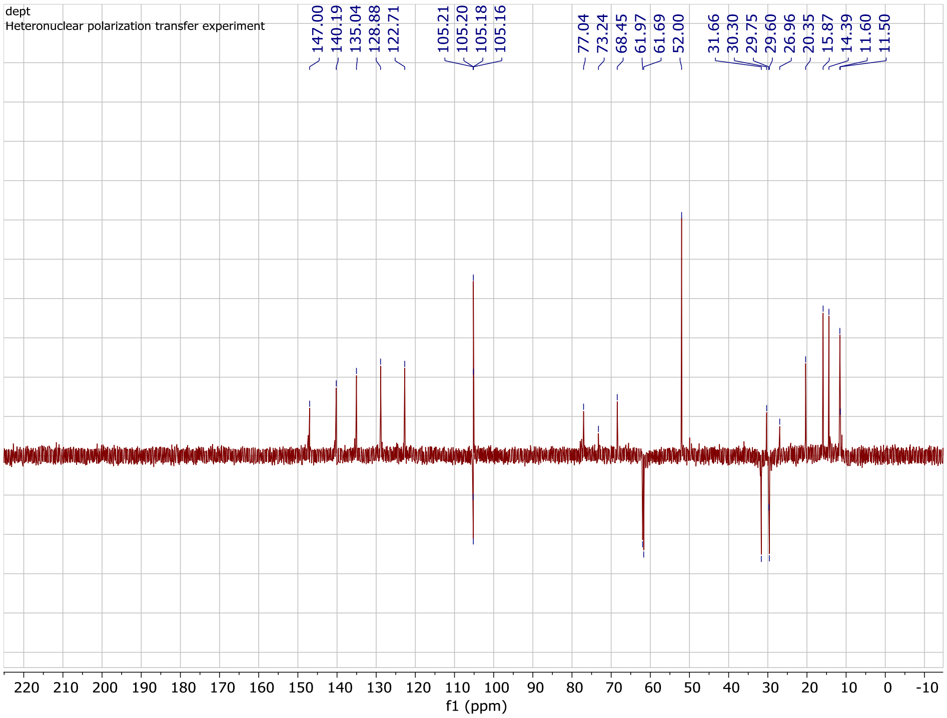

Figure S 27: ^{13}C NMR Spectrum of compound **7** in $CDCl_3$/methanol-*d*$_4$ (4:1)

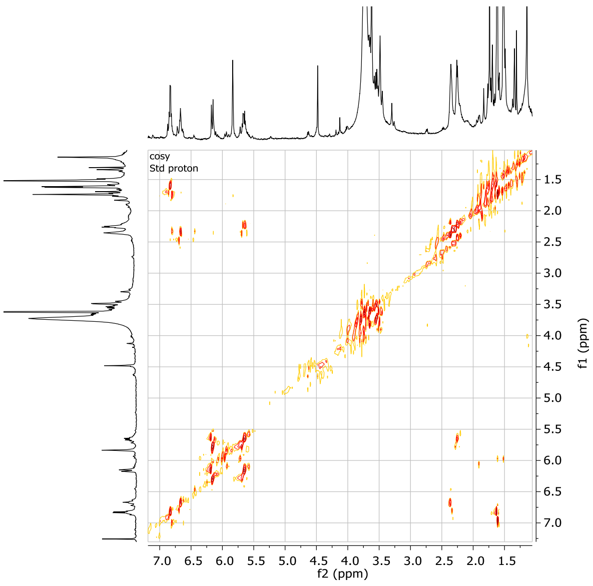

Figure S 28: COSY Spectrum of compound **7** in $CDCl_3$/methanol-d_4 (4:1)

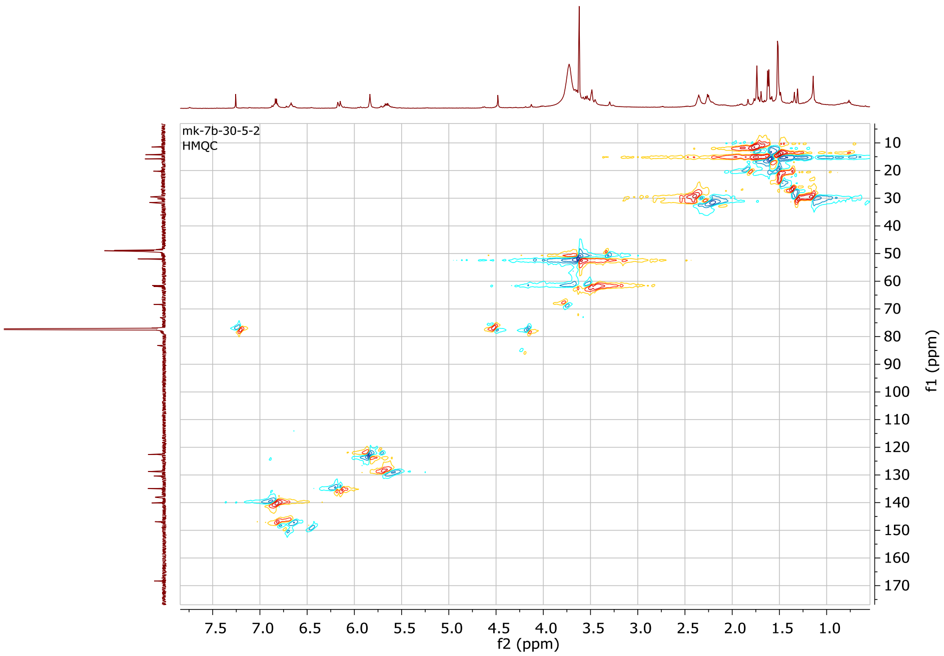

Figure S 29: HMQC Spectrum of compound **7** in $CDCl_3$/methanol-d_4 (4:1)

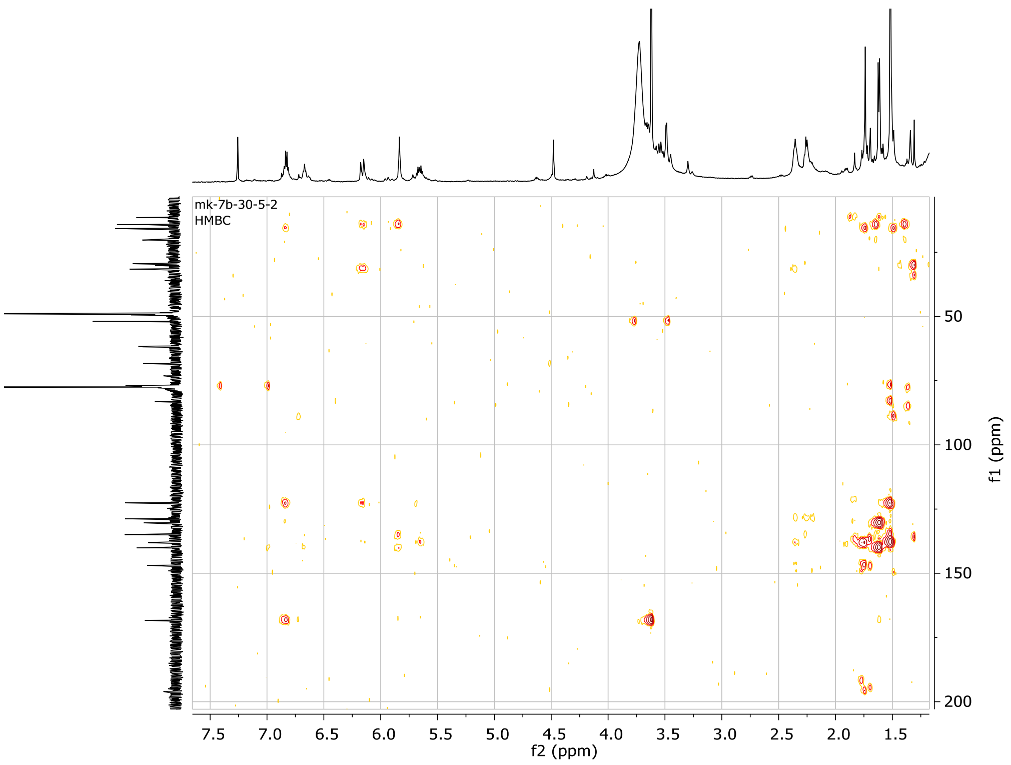

Figure S 30: HMBC Spectrum of compound **7** in $CDCl_3$/methanol-d_4 (4:1)

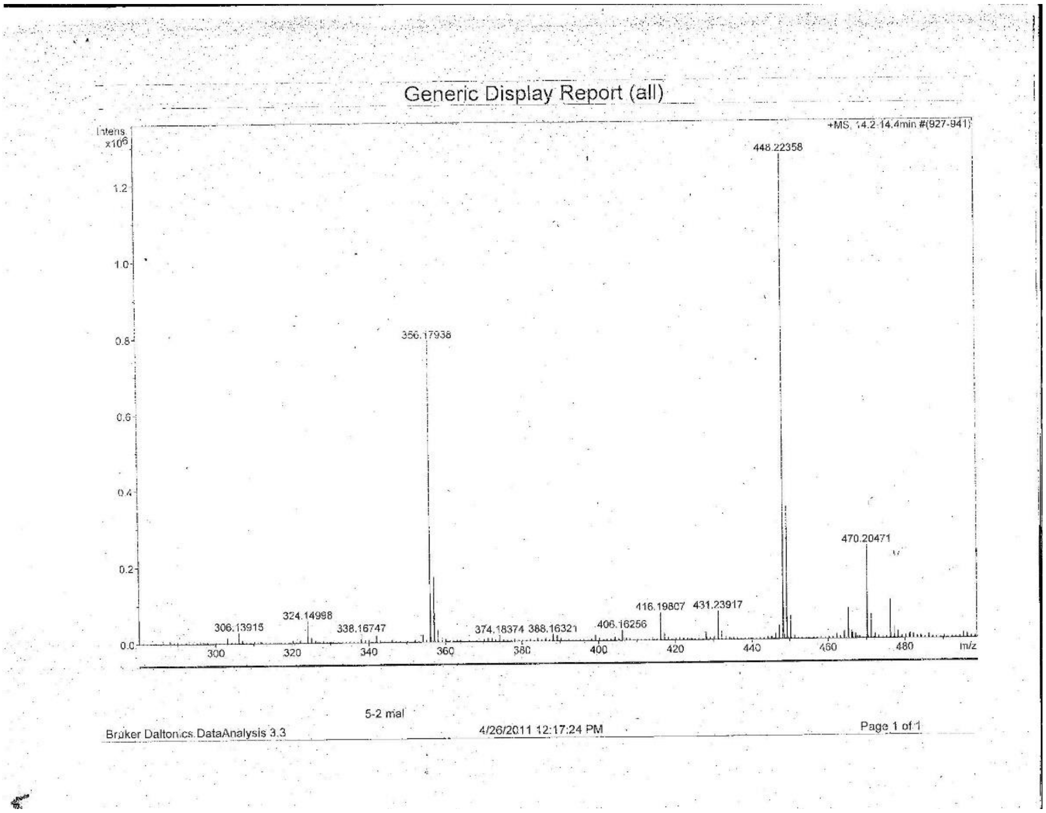

Figure S 31: HRMS Data of Compound **7**

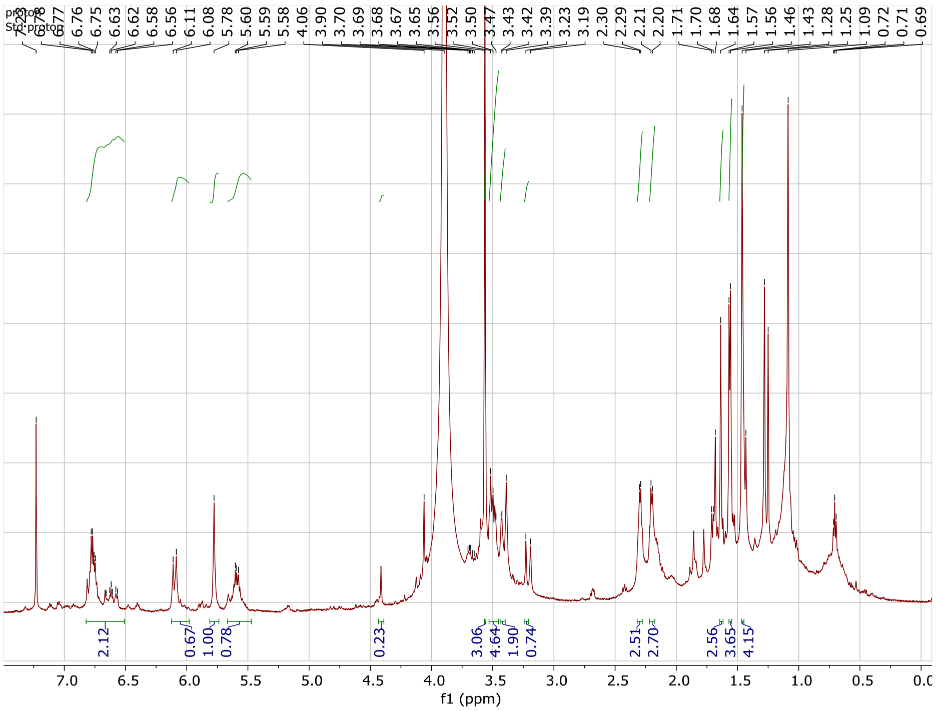

Figure S 32: ^{1}H NMR Spectrum of compound **8** in $CDCl_3$/methanol-d_4 (1:1)

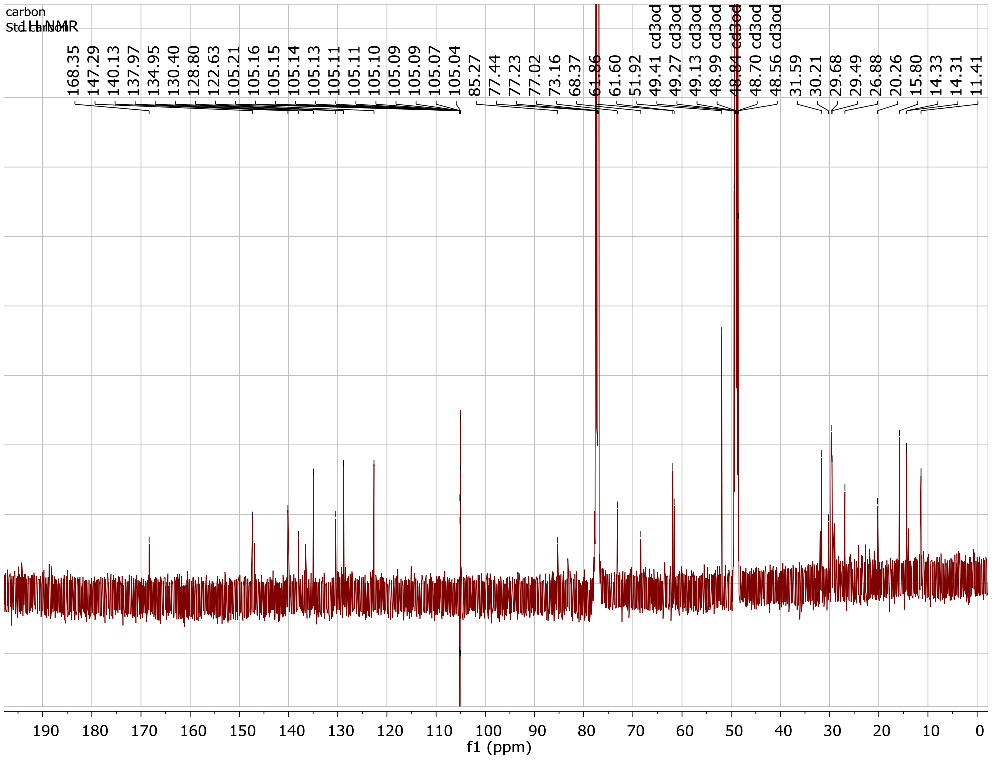

Figure S 33: ^{13}C NMR Spectrum of compound **8** in $CDCl_3$/methanol-d_4 (1:1)

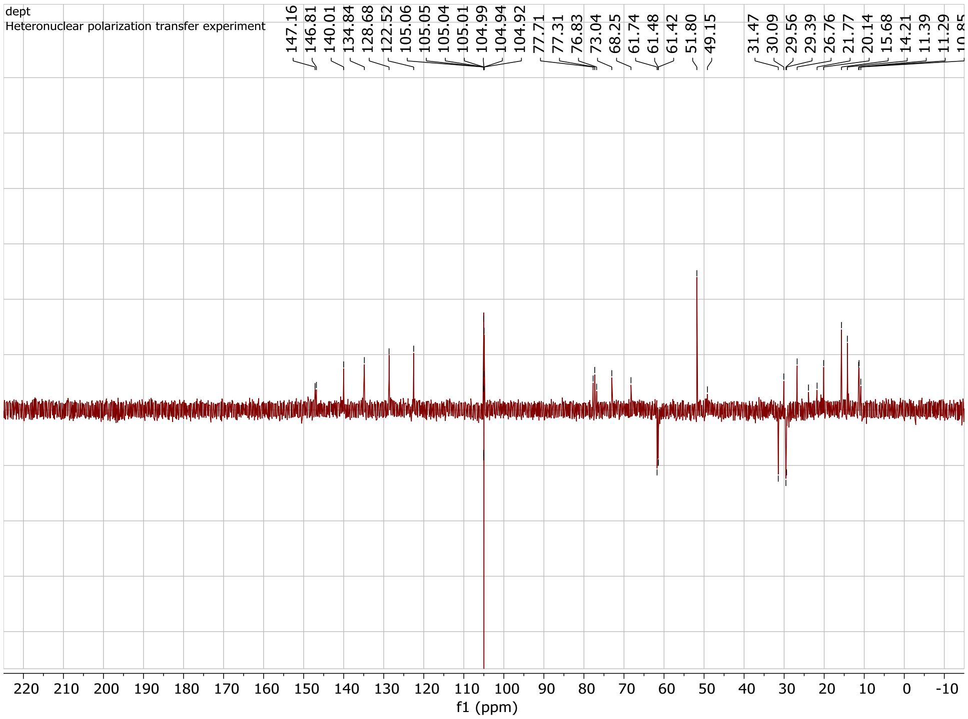

Figure S 34: DEPT Spectrum of compound **8** in $CDCl_3$/methanol-d_4 (1:1)

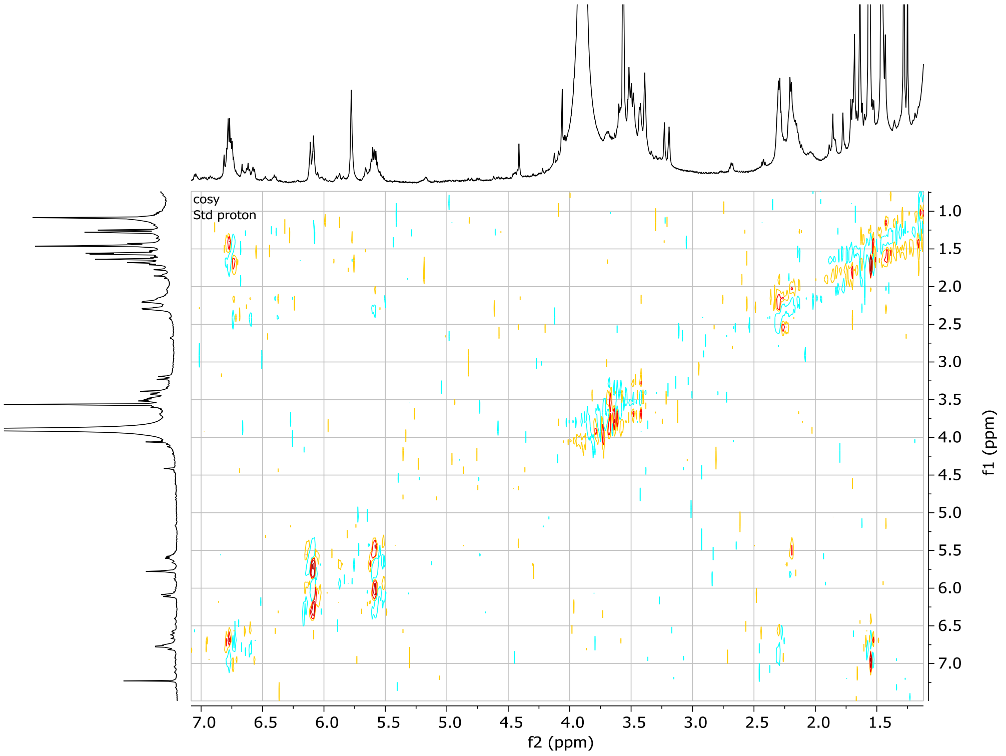

Figure S 35: COSY Spectrum of compound **8** in $CDCl_3$/methanol-d_4 (1:1)

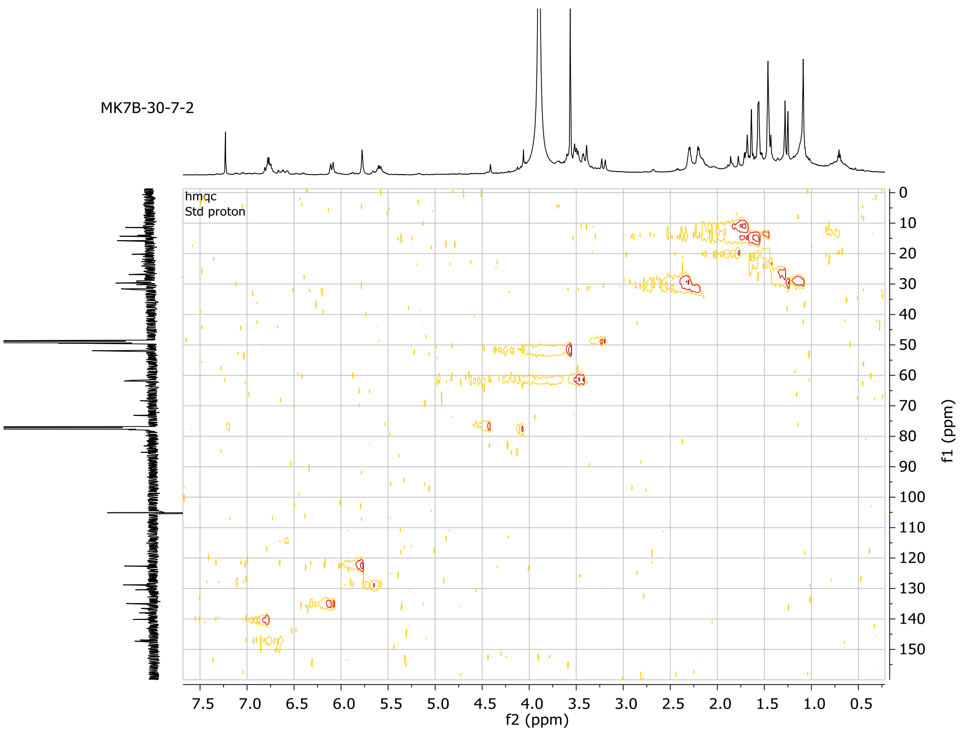

Figure S 36: HMQC Spectrum of compound **8** in $CDCl_3$/methanol-d_4 (1:1)

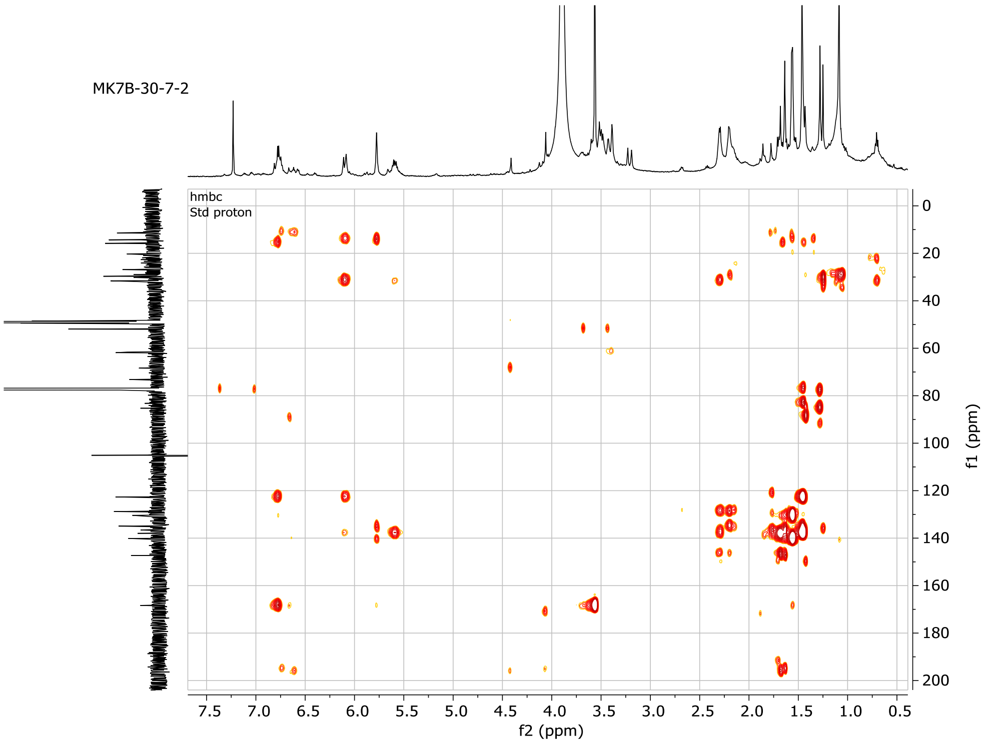

Figure S 37: HMBC Spectrum of compound **8** in $CDCl_3$/methanol-d_4 (1:1)

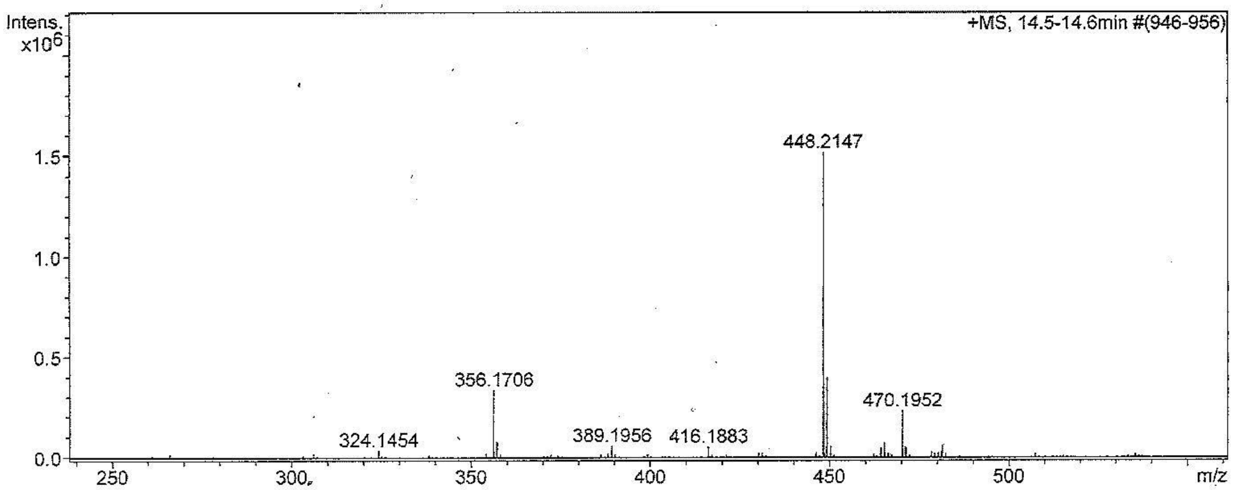

Figure S 38: HRMS Data of Compound **8**

Table S1: Best nineteen hits 100% sequence identity

MG282093.1	*Botryosphaeria dothidea* isolate sdxf6
KF551232.1	*Botryosphaeria dothidea* strain LBSX-1
JX275795.1	*Botryosphaeria dothidea* strain SCCDHBJ01S
JQ936677.1	*Botryosphaeria dothidea* strain Shi Liu Branch
JF441086.1	*Botryosphaeria dothidea* strain GS-02s-6
HQ660460.1	*Botryosphaeria dothidea* isolate XNHG-12WR
AB454278.1	*Botryosphaeria dothidea* isolate MUCC0039
MF409167.1	*Botryosphaeria dothidea* strain MFLUCC_17_0961
KU686880.1	*Botryosphaeria dothidea* strain MFLUCC_16_0092
KU686879.1	*Botryosphaeria dothidea* strain MFLUCC_16_0087
KU686878.1	*Botryosphaeria dothidea* strain MFLUCC_16_0085
KU686877.1	*Botryosphaeria dothidea* strain MFLUCC_16_0076
JX867231.1	*Botryosphaeria dothidea* strain JL-31
JF441084.1	*Botryosphaeria dothidea* strain HNZs-1
AB645744.1	*Botryosphaeria dothidea* strain FFPRI411066
EU441944.1	*Botryosphaeria dothidea* strain SDAU07080
KM435312.1	*Botryosphaeria dothidea* isolate Hen 1
KJ530706.1	*Botryosphaeria dothidea* strain YDJ-08
FJ550211.1	*Botryosphaeria dothidea* strain SDAU-08-73

Table S2: ITS sequences of taxa of the Botryosphaeriaceae used for alignment

H133917.1	*Aplosporella ginkgonis* strain CFCC 52443
MH133913.1	*Aplosporella javeedii* strain CFCC 52439
KM030581.1	*Aplosporella javeedii* strain CFCC 89659
EF564375.1	*Aplosporella prunicola* strain STE-U 6326
EF564376.1	*Aplosporella prunicola* strain STE-U 6327
KY173388.1	*Aplosporella sophorae* strain CPC 29688
KX235306.1	*Barriopsis archontophoenicis* strain MFL UCC 14-1164
KF766149.1	*Barriopsis fusca* strain CBS 174.16
FJ919663.1	*Barriopsis iraniana* strain IRAN1448C
FJ919667.1	*Barriopsis iraniana* strain IRAN1450C
MG282093.1	*Botryosphaeria dothidea* isolate sdxf6
KF551232.1	*Botryosphaeria dothidea* strain LBSX-1
JX275795.1	*Botryosphaeria dothidea* strain SCCDHBJ01S
GU251174.1	*Dichomera eucalypti* strain PD294
AY744379.1	*Dichomera saubinettii* strain CBS990 70
GU251222.1	*Dichomera versiformis* strain PD295
AY744376.1	*Dichomera versiformis* strain WAC15208
GQ923860.1	*Diplodia malorum* strain CAP266
EF445346.1	*Diplodia mutila* strain STE-U 5824
EF445341.1	*Diplodia pinea* strain STE-U 5809
KF500479.1	*Diplodia quercivora* strain PL1345
MH863064.1	*Dothidotthia symphoricarpi*
KX228269.1	*Dothiorella acacicola* strain CPC 26349
KT240287.1	*Dothiorella eriobotryae* isolate BN-81
KY797637.1	*Dothiorella guttulata* strain MFLUCC 17-0242
FJ481601.1	*Dothiorella iberica* strain B33
EF445361.1	*Dothiorella viticola* strain STE-U 5831
EF445360.1	*Dothiorella viticola* strain STE-U 6139
AY819725.1	*Fusicoccum arbuti* isolate UAMH 6800
AY819724.1	*Fusicoccum arbuti* isolate UW13
AY853214.1	*Fusicoccum quercus* strain CBS 115298
MH290868.1	*Guignardia ardisiae* strain VDL2
MK299126.1	*Guignardia cryptomeriae* isolate LWU 27
MH935065.1	*Guignardia gaultheriae* isolate SO1 T29 L3A 2
FJ538334.1	*Guignardia musicola* strain CBS 123405
KF206174.1	*Guignardia rhodorae* strain CBS 901.69
JN692544.1	*Guignardia sansevieriae* CBS:120428
MH396638.1	*Guignardia* sp. strain PM011
KT151806.1	*Lasiodiplodia brasiliense* strain IBL415

KT154762.1	*Lasiodiplodia caatinguensis* strain IBL40
KJ638320.1	*Lasiodiplodia exigua* strain BL186
KP822975.1	*Lasiodiplodia iranensis* strain CERC2032
EU520120.1	*Macrophoma sophoricola* isolate NW623
KP017858.1	*Macrophoma* sp. IK-2015 strain IR-3Sa-2-3-3
KJ183691.1	*Macrophoma* sp. LYZ2007
AB541378.1	*Macrophoma sugi* strain: M1-4
KP179222.1	*Macrophoma theicola* strain UPA-62
KY680345.1	*Macrophomina phaseolina* strain CCTU1656
KU058940.1	*Macrophomina phaseolina* strain CDA1100
KF951718.1	*Macrophomina phaseolina* strain CPC 21421
KU058950.1	*Macrophomina pseudophaseolina* strain CD A1110
AY194467.1	*Nattrassia mangiferae* strain CMW 10332
KP165429.1	*Neodeightonia licuriensis* strain COAD1780
KU940110.1	*Neodeightonia microspora* strain MFLUCC 11-0483
MK203813.1	*Neodeightonia palmicola* isolate FAFU002
JX456475.1	*Neodeightonia phoenicum* isolate FK6
KX646359.1	*Neodeightonia rattanicola* voucher MFLUCC15-0319
EU375516.1	*Neofusicoccum australe* isolate JL619
JX513628.1	*Neofusicoccum brasiliense* strain CMM128
KP860870.1	*Neofusicoccum kwambonambiense* strain CMW42349
KP860867.1	*Neofusicoccum luteum* strain CMW41220
KX464197.1	*Neofusicoccum parvum* culture CBS:257.77
KX464196.1	*Neofusicoccum* sp. CBS 118100 culture-CBS:118100
EF445351.1	*Neofusicoccum vitifusiforme* strain STE-U 5910
MH791087.1	*Neoscytalidium dimidiatum* isolate A1F
EU597630.1	*Neoscytalidium dimidiatum* strain MCCL 340001
EF570499.1	*Neoscytalidium dimidiatum* strain UAMH 10614
JQ927342.1	*Neoscytalidium hyalinum* strain CMM3616
GU172400.1	*Neoscytalidium novaehollandiae* strain WAC13275
AY343384.1	*Otthia spiraeae* strain imi63581
EU520165.1	*Phaeobotryon cupressi* isolate NW568B
EU673336.1	*Phaeobotryon mamane* strain CPC 12445
NR 155669.1	*Phaeobotryon negundinis* MFLUCC 15-0436
KM030585.1	*Phaeobotryon rhois* strain CFCC 89663
KF766146.1	*Phaeosphaeria ammophilae* strain CBS 114595
KY775577.1	*Phaeosphaeriaceae* sp. AM-2017a voucher MFLU 17-0498
MH857214.1	*Phomatosphaeropsis pinicola*
EU683671.1	*Phyllosticta capitalensis* strain 1095(12)
FJ538339.1	*Phyllosticta capitalensis* strain CBS 117118

FJ538340.1	*Phyllosticta capitalensis* strain CBS 119720
EF585520.1	*Pseudofusicoccum adansoniae* strain BRIP 19782
NR 155641.1	*Pseudofusicoccum artocarpi* CPC 22796
EU144056.1	*Pseudofusicoccum kimberleyense* strain CBS122059
MG386028.1	*Saccharata acaciae* culture CBS:143167
NR 155851.1	*Saccharata banksiae* CPC 27698
EU552129.1	*Saccharata capensis* culture-collection CBS:122694
KY173450.1	*Saccharata daviesiae* strain CPC 29174
KY173454.1	*Saccharata hakeae* strain CPC 29250
HM107423.1	*Thyrostroma carpophilum* isolate edith
KC815898.1	*Thyrostroma carpophilum* isolate WcB7
KX228249.1	*Thyrostroma compactum* strain CBS 487.71
